# Performance of AI in Predicting the Progression of Gestational Diabetes to Type 2 Diabetes: Systematic Review and Meta-Analysis

**DOI:** 10.2196/87882

**Published:** 2026-07-09

**Authors:** Alaa Abd-alrazaq, Shahira Padinharepattel Mohamed, Mohannad Alajlani, Aliya Tabassum, José Manuel Ordóñez-Mena, Shehel Yoosuf, Mais Alkhateeb, Arfan Ahmed, Mohammed Bashir, Junaid Qadir, Ali AlSanousi, Javaid Sheikh

**Affiliations:** 1 AI Center for Precision Health, Weill Cornell Medicine-Qatar, Qatar Foundation, A031, Doha, Doha, 000, Qatar, 974 453456653; 2 Department of Biomedical Sciences, College of Health Sciences, Qatar University, Doha, Qatar; 3 Division of Information and Computing Technology, College of Science and Engineering, Hamad Bin Khalifa University, Qatar Foundation, Doha, Qatar; 4 Institute of Digital Healthcare, WMG, University of Warwick, Warwick, England, United Kingdom; 5 Computer Science and Engineering Department, College of Engineering, Qatar University, Doha, Qatar; 6 Nuffield Department of Primary Care Health Sciences, University of Oxford, Oxford, United Kingdom; 7 Qatar Metabolic Institute, Hamad Medical Corporation, Doha, Qatar; 8 Clinical Information Systems Department, Hamad Medical Corporation, Doha, Qatar

**Keywords:** diabetes mellitus, gestational diabetes, prediabetes, artificial intelligence, machine learning, systematic review, meta-analysis

## Abstract

**Background:**

Gestational diabetes mellitus (GDM) significantly increases the risk of developing type 2 diabetes mellitus (T2DM) post partum, with up to half of affected women progressing within a decade. Early identification of high-risk individuals is critical for implementing preventive interventions. Artificial intelligence (AI) offers enhanced predictive capabilities that can substantially enhance the prevention of postpartum diabetes.

**Objective:**

This systematic review and meta-analysis aimed to evaluate the performance of AI models in predicting the progression from GDM to T2DM or prediabetes.

**Methods:**

A total of 7 databases (MEDLINE, Embase, Scopus, Web of Science, IEEE Xplore, ACM Digital Library, and Google Scholar) were systematically searched from inception through September 12, 2025, supplemented by backward and forward reference screening and biweekly alerts to capture newly published studies. This review included peer-reviewed English-language studies that applied AI algorithms to predict T2DM or prediabetes among women with previous GDM. Eligible studies focused on human participants; reported performance metrics (eg, accuracy, sensitivity, and specificity); and excluded non-AI models, animal studies, reviews, protocols, abstracts, and non-English publications. Moreover, 2 reviewers independently conducted study selection, data extraction, and risk of bias assessment using the PROBAST (Prediction Model Risk of Bias Assessment Tool)+AI tool. Pooled estimates were computed using random-effects meta-analysis models.

**Results:**

In total, 10 studies met the inclusion criteria, of which 8 were eligible for meta-analysis. The reviewed studies spanned from 2011 to 2025 and were conducted across 7 countries, predominantly in the United States (3/10, 30%). Most publications were journal articles (9/10, 90%), and retrospective designs (6/10, 60%) were slightly more common than prospective designs (4/10, 40%). AI models demonstrated high predictive performance for T2DM, with pooled accuracy of 0.85 (95% CI 0.79‐0.90; prediction interval [PI] 0.64‐0.98), sensitivity of 0.89 (95% CI 0.81‐0.95; PI 0.63‐1.00), specificity of 0.88 (95% CI 0.81‐0.93; PI 0.67‐0.99), *F*_1_-score of 0.80 (95% CI 0.75‐0.85; PI 0.68‐0.93), and area under the curve of 0.86 (95% CI 0.77‐0.91; PI 0.54‐0.97). However, AI performance for prediabetes prediction was modest (area under the curve=0.69, 95% CI 0.60‐0.77). Subgroup analyses showed that random forest, decision tree, logistic regression, and naïve Bayes models performed comparably. Fasting plasma glucose and BMI were the most identified significant predictors in the included studies.

**Conclusions:**

AI models show potential in predicting T2DM after GDM. However, evidence remains limited by small sample sizes, high heterogeneity, lack of external validation, and high risk of bias. Our findings have important implications for digital health, supporting the integration of AI-driven risk prediction into electronic health record systems and postpartum care pathways to enable early identification, targeted prevention, and improved long-term outcomes. Future research should use large, diverse cohorts, integrate multidimensional data, adopt standardized reporting frameworks, and encourage open-access data sharing.

## Introduction

### Background

Gestational diabetes mellitus (GDM) is an increasingly common complication of pregnancy that is characterized by elevated blood glucose levels and glucose intolerance. While it often resolves naturally after childbirth, women with a history of GDM have an approximately tenfold higher risk of developing type 2 diabetes mellitus (T2DM) compared with those with normoglycemic pregnancies [1]. The progression to T2DM can be rapid, with estimates indicating that 30%‐50% of women with previous GDM develop T2DM within 5 to 10 years post partum [2-4]. A meta-analysis including 170,139 women estimated that the annual rate of progression from GDM to T2DM was 9.6% [5]. These findings highlight GDM as a significant predictor of T2DM in young women [6]. Therefore, this population requires structured follow-up, including annual T2DM screening and targeted prevention programs to reduce their future risk of developing T2DM.

The public health implications of GDM are growing. Global prevalence continues to rise, driven by multiple factors, including lifestyle changes, increasing maternal age, and higher obesity rates. Importantly, improved screening practices have also contributed substantially to this trend. For example, data from Qatar show that shifting from a single-center universal screening approach to national universal screening increased the documented prevalence of GDM from approximately 21% to around 32% [7,8]. As more women are diagnosed, the burden of postpartum care, prevention, and long-term metabolic follow-up intensifies for health care systems.

The susceptibility of women with GDM to transition into patients with T2DM not only poses serious long-term health consequences for the individual but also represents a growing economic and public health burden [9,10]. Early detection and timely intervention are therefore critical to reducing disease incidence and mitigating associated health care costs [11]. Yet, despite clear guidelines, postpartum screening remains highly suboptimal. Compliance with the ADA recommendations is reported to be as low as 16%‐19% [12,13]. This poor adherence is often attributed to logistical difficulties (eg, administering the test), failure to attend follow-ups, and a misguided perception of low risk among patients [12,13].

Given the increasing prevalence of GDM and, by extension, the enlarging at-risk population, there is a pressing need for tools that can stratify women into high, intermediate, and low-risk categories for future progression to T2DM. Effective stratification enables targeted interventions and more efficient screening programs, improving preventive efficacy while reducing unnecessary costs. In this context, tools that can accurately predict progression to T2DM (high-risk) and prediabetes (intermediate-risk) are essential for optimizing long-term management.

Artificial intelligence (AI) methods have been established as a powerful tool for predictive population risk stratification and improving patient outcomes through enhanced prognosis accuracy. Sophisticated AI techniques can be used to analyze comprehensive patient data (eg, demographics, clinical histories, diagnostics, and therapeutic outcomes). By discerning patterns and correlations within these datasets, AI algorithms (eg, random forest [RF], decision tree [DT], logistic regression [LogReg], multilayer perceptron, naïve Bayes [NB], extreme gradient boosting [XGBoost]) construct models that predict patient outcomes with superior accuracy compared with conventional statistical approaches, such as Cox proportional hazards models and traditional LogReg built without AI-based feature optimization. AI models represent the algorithmic modeling culture, emphasizing predictive performance and pattern discovery, while traditional regression models represent the data modeling culture, emphasizing explanation and interpretability [14]. This data-driven process enables the identification of novel risk factors and complex interactions that may not be apparent through human observation or traditional methods, ultimately facilitating a more personalized and proactive approach to patient care. Thus, in recent years, AI has emerged as a promising tool for predicting the progression from GDM to T2DM.

### Research Problem and Aim

Several studies have applied AI-based methods to predict the progression from GDM to T2DM. Moreover, 2 recent systematic reviews have attempted to compile and summarize the findings from these studies [15,16]. However, both reviews exhibit methodological limitations that underscore the need for a more comprehensive and rigorous evaluation. Both reviews did not search important databases. Specifically, the review by Elfadel Magboul et al [15] did not search Embase, Google Scholar, and the ACM Digital Library, while the review by Zhao et al [16] did not search Scopus, IEEE Xplore, and the ACM Digital Library. In addition, one of the reviews did not conduct a meta-analysis and was not registered in any systematic review registry [15], while the other did not perform backward and forward reference list checking [16]. Furthermore, both reviews included the same 13 studies; however, 6 of these were erroneously included, as they either used traditional statistical techniques instead of AI-based models [17-20] or focused on outcomes unrelated to the prediction of GDM progression to T2DM [21,22]. Finally, both reviews used PROBAST (Prediction Model Risk of Bias Assessment Tool) [23], rather than the more relevant and recently proposed PROBAST-AI version [24], which has been specifically developed to assess bias in AI model studies. These combined limitations undermine the validity of the existing literature and highlight the need for a new, methodologically sound systematic review. This review aimed to evaluate the performance of AI in predicting the transition from GDM to T2DM, while addressing the methodological shortcomings of previous reviews.

## Methods

### Overview

This review was conducted and reported in line with the guidelines provided in the PRISMA-DTA (Preferred Reporting Items for Systematic Reviews and Meta-Analyses - Extension for Diagnostic Test Accuracy) [25]. Multimedia Appendix 1 highlights this review’s PRISMA-DTA Checklist. Its protocol has been registered with the PROSPERO (International Prospective Register of Systematic Reviews; CRD420251163311).

### Search Strategy

The search strategy was conducted and reported in accordance with the PRISMA-S (Preferred Reporting Items for Systematic Reviews and Meta-Analyses literature search extension) guidelines [26]. On September 12, 2025, we conducted a comprehensive search across several electronic databases, such as MEDLINE (via Ovid), Embase (via Ovid), Scopus, Web of Science, ACM Digital Library, IEEE Xplore, and Google Scholar. An automatic alert was scheduled to rerun the search every 2 weeks for 6 months, with the final update conducted on March 11, 2026. Because Google Scholar produced an exceptionally large number of results, only the first 100 entries (10 pages) were screened [27]. To ensure completeness, we also performed backward citation checking (reviewing reference lists of included studies) and forward citation checking (examining studies that cited the included papers) [27]. We did not use any of the following information sources: study registries, online resources and browsing, and contacting authors or experts.

The search strategy integrated three groups of keywords: (1) AI-related terms (eg, “artificial intelligence,” “artificial intelligence,” “machine learning,” and “deep learning”), (2) terms related to GDM (eg, gestational diabet*, pregnancy-induced diabet*, and GDM), and (3) terms referring to T2DM (eg, “type 2 diabet*” and “ketosis-resistant diabet*”). Boolean operators “OR” and “AND” were used to combine terms within and across categories, respectively [27]. The search was limited to the English language. The search strategy was not formally peer-reviewed due to resource constraints; however, it was developed iteratively and validated through pilot searches and consultation among the research team. This review did not use published search filters or adapted search strategies from other literature reviews. The complete search strings used for each database are provided in Multimedia Appendix 2.

### Study Eligibility Criteria

This review included studies that used AI algorithms to detect the progression of GDM to T2DM or prediabetes. Research articles deemed suitable for inclusion were those focusing on women diagnosed with GDM, regardless of age, ethnicity, parity, or other characteristics. Studies involving animals were excluded. To qualify for inclusion, studies had to evaluate the performance of AI algorithms in predicting the occurrence of T2DM or prediabetes among patients with a history of GDM, regardless of the test or criteria used for diagnosing GDM, T2DM, and prediabetes. Eligible studies were required to provide a confusion matrix and/or performance metrics (eg, accuracy, sensitivity, and specificity). Studies that used AI solely to predict T2DM irrespective of previous GDM diagnosis were excluded. Furthermore, studies using AI for other purposes (eg, predicting postpartum diabetes screening attendance or maternal or fetal outcomes) were also excluded. In addition, studies that developed predictive models not based on AI algorithms were excluded (eg, Cox regression for survival analysis). For the purpose of this review, AI models were operationally defined as data-driven algorithms capable of automated pattern recognition and prediction, including nonlinear models (eg, support vector machines with kernels), ensemble methods (eg, RF and XGBoost), neural networks, and other AI approaches. Linear models such as LogReg were considered AI-based only when implemented within an AI framework (eg, incorporating automated feature selection, regularization, or cross-validation). Conventional regression models relying solely on manually specified predictors without AI components were excluded. Only peer-reviewed journal articles, conference papers, and dissertations were included, with no restrictions on study setting, design, reference standard (ie, ground truth), year of publication, country, or follow-up duration. However, papers not published in English or categorized as editorials, reviews, protocols, posters, conference abstracts, or research highlights were excluded from consideration.

### Study Selection

The study selection process followed 3 main stages [27]. First, duplicate records were removed using EndNote 21 (Clarivate). Next, 2 reviewers (SPM and M Alajlani) independently screened the titles and abstracts of the remaining papers to determine eligibility. Finally, both reviewers separately evaluated the full-text articles. Any disagreements were discussed and resolved with input from a third reviewer (AAa). Interreviewer agreement was high, with κ scores of 0.81 for the title or abstract screening and 0.88 for the full-text assessment.

### Data Extraction

To design and refine the data extraction form (Multimedia Appendix 3), 2 studies were initially piloted [27]. Moreover, 2 reviewers (SPM and M Alajlani) then independently extracted information using Microsoft Excel, including study metadata, participant characteristics, and details of the AI models used. Additionally, we recorded each algorithm’s best-reported performance across accuracy, sensitivity, specificity, *F*_1_-score, and area under the curve (AUC). When confusion matrices were available, we computed all relevant performance metrics (eg, accuracy, sensitivity, and specificity). If such information was missing, we attempted to obtain it by contacting the first and corresponding authors [27]. Any inconsistencies between reviewers were resolved by a third reviewer (AAa).

### Risk of Bias and Applicability Appraisal

To assess the risk of bias and applicability of the included studies, we used a recent tool called PROBAST+AI [24], built upon PROBAST [23], with additional elements specifically designed to capture methodological nuances related to AI model development and validation.

The PROBAST+AI tool consists of 4 domains—participants, predictors, outcome, and analysis. Each domain includes multiple signaling questions designed to guide judgments about potential bias and the applicability of study findings to the review question [24]. Responses to each question were rated as yes (Y), probably yes (PY), no (N), probably no (PN), or no information (NI) [24]. Ratings of Y and PY indicate low risk of bias, whereas N and PN indicate high risk. NI was used only when information was insufficient to form a judgment [24]. The first 3 domains (participants, predictors, and outcome) were also assessed for applicability concerns, evaluating whether the study’s population, predictor definitions, and outcome measurements aligned with the objectives of our review [24]. An overall judgment of risk of bias was assigned as follows [24]: (1) low risk of bias, if all 4 domains were rated low risk; (2) high risk of bias, if any domain was rated high risk; and (3) unclear risk of bias, if at least 1 domain was unclear and none were high risk [24]. Applicability concerns were summarized similarly [24].

To ensure consistency and reliability, 3 reviewers (SPM, M Alajlani, and AT) independently assessed each study. Discrepancies in ratings were resolved through discussion and consensus. Before starting the process, a pilot assessment involving 2 studies was conducted to refine the criteria and ensure interreviewer agreement. The final assessment tool is provided in Multimedia Appendix 4. To enhance methodological transparency, we provide a supplementary mapping of key CHARMS (Checklist for Critical Appraisal and Data Extraction for Systematic Reviews of Prediction Modelling Studies) checklist domains to the PROBAST+AI framework (Multimedia Appendix 4), illustrating how core prediction model elements were captured within our assessment approach.

### Data Synthesis

For quantitative synthesis, we selected 1 model per study to avoid double-counting participants and violating statistical independence assumptions. When multiple models were reported, the primary analysis extracted the best-performing model as defined by the study authors, typically based on the primary performance metric emphasized in the study (eg, AUC, accuracy, or *F*_1_-score). While this approach reflects how studies typically present their final or recommended predictive model, it may introduce systematic optimism, as performance estimates can be influenced by internal model selection, hyperparameter tuning, and differences in validation strategies. To assess the potential magnitude and direction of this optimism, we conducted additional meta-analyses using the worst-performing reported model within each study. These analyses were designed to provide a more conservative lower-bound estimate of predictive performance and to evaluate the robustness of pooled findings.

We conducted meta-analyses using random-effects models, with between-study variance (τ²) estimated using the restricted maximum likelihood (REML) method [28]. Specifically, for meta-analyses of accuracy, sensitivity, and specificity, we used the function for meta-analysis of proportions within the *meta* package [29,30] using the numerator and denominator as reported by the included studies. Proportions were transformed using the Freeman-Tukey double-arcsine transformation for variance stabilization [31-33]. We used the generic inverse variance weighting method for pooling untransformed *F*_1_-scores across studies. When either the *F*_1_-score or its SE were not reported, we calculated them using the confusion matrix cell counts and methods described elsewhere [34,35]. We pooled the study-specific estimates of AUC across studies using the generic inverse variance weighting method with the *meta* package after first converting study-specific estimates into log odds and estimating their SEs using the methods in the *metamisc* package [36]. When applicable, subgroup meta-analyses were conducted to explore how AI algorithms (eg, RF, DT, and XGBoost) might influence the performance of AI in predicting the transition from.

Although sensitivity and specificity are inherently correlated through classification thresholds, we pooled these metrics separately using random-effects models. This approach was adopted for several reasons. First, only a limited number of studies reported both sensitivity and specificity. Second, many studies did not provide sufficient data (eg, full contingency tables or threshold-specific parameters) to enable joint estimation of these metrics. Third, substantial methodological heterogeneity was observed across studies, including differences in AI algorithms, prediction horizons, validation strategies, and predictor sets. These reasons precluded the use of hierarchical diagnostic meta-analysis methods, such as the bivariate model or the hierarchical summary receiver operating characteristic model. Therefore, separate random-effects pooling of performance metrics was considered a pragmatic and appropriate approach to summarize average model performance across heterogeneous studies.

To evaluate the consistency of findings across studies (heterogeneity), we applied 3 statistical methods [27]. First, we used the Cochrane Q test, which determines whether the variability in study results could be explained by chance alone [28]. A *P* value below .05 suggests significant heterogeneity, indicating that the differences between studies exceed what would be expected randomly. Second, we calculated the *I*^2^ statistic to measure the proportion of total variation attributable to true differences across studies rather than random error [28]. According to established thresholds, heterogeneity was interpreted as negligible (0%‐40%), moderate (30%‐60%), substantial (50%‐90%), or considerable (75%‐100%) [28]. Third, we estimated the between-study variance (τ²) using the REML approach [28]. For meta-analyses with 3 or more studies (overall and within subgroups), we computed prediction intervals (PIs) using the method by Nagashima et al [37] to demonstrate the variability in the AI models’ performance across various observed settings and in the future.

We assessed publication bias using both visual and statistical approaches. Funnel plots were generated for each performance metric (accuracy, sensitivity, specificity, *F*_1_-score, and AUC) to evaluate the presence of small-study effects. Visual asymmetry was assessed qualitatively. In addition, the Egger regression test was performed to statistically evaluate funnel plot asymmetry, with *P*<.05 indicating potential publication bias. All analyses (ie, meta-analysis, heterogeneity, and publication bias) were carried out using R (version 4.5.2; R Core Team).

### Assessment of Overlap Between Reviews

To assess the degree of overlap between primary studies included in this review and those in existing systematic reviews, we calculated the corrected covered area (CCA), a validated metric for quantifying overlap in reviews [38]. The CCA was computed across all reviews and through pairwise comparisons to provide a more granular assessment of shared evidence, with higher values indicating greater redundancy in included primary studies. The CCA was calculated using the standard formula [38]:


CCA=(N−r)/(rc−r)

where N is the total number of included study occurrences across reviews, r is the number of unique primary studies, and c is the number of reviews.

### Deviations From Protocol

Although this review followed a predefined protocol registered in PROSPERO (CRD420251163311), several refinements were introduced to enhance analytical rigor and interpretability. Specifically, PIs were not originally specified in the protocol but were subsequently calculated and reported for meta-analyses including 3 or more studies. This addition was made to provide a more comprehensive assessment of between-study variability and to better reflect the expected range of effect estimates in future settings. In addition, while the protocol specified the use of DerSimonian-Laird random-effects models, the final analyses used the REML method to estimate between-study variance, as this approach has been shown to provide more robust and less biased estimates, particularly in the presence of heterogeneity [28]. Additional performance metrics, including the *F*_1_-score and AUC, were also synthesized beyond the originally planned accuracy, sensitivity, and specificity. Furthermore, sensitivity analyses were conducted by comparing best-performing and worst-performing models within each study to provide both upper- and lower-bound estimates of predictive performance; this approach was not prespecified in the protocol. Additionally, publication bias was assessed using funnel plots and the Egger regression test, although this was not originally planned in the protocol. Finally, we conducted an assessment of overlap between included studies and previous systematic reviews using the CCA, which was not specified in the original protocol but was added to improve methodological transparency.

## Results

### Search Results

**Figure 1.**
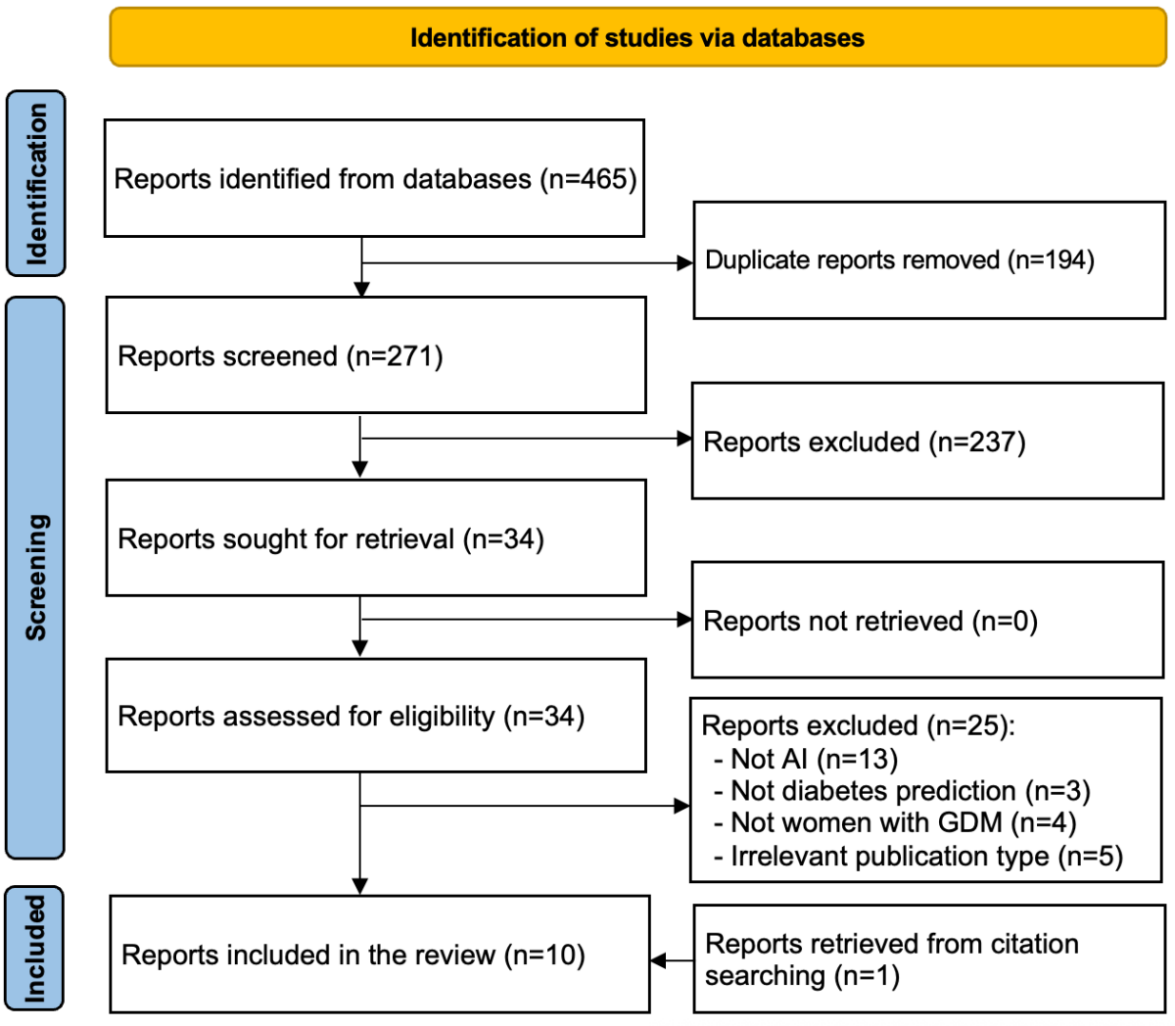
Flow chart of the study selection process. The figure summarizes the identification, screening, eligibility, and inclusion of studies from multiple databases (searched up to September 2025), including removal of duplicates, title/abstract screening, full-text assessment, and reasons for exclusion. A total of 465 records were identified, 271 were screened after duplicate removal, and 10 studies were ultimately included. GDM: gestational diabetes mellitus.

As illustrated in Figure 1, our database search yielded 465 citations. After removing 194 duplicates using EndNote 21, a total of 271 unique records remained. Screening the titles and abstracts led to the exclusion of 237 studies. The full texts of the remaining 34 articles were then reviewed in detail, and 25 were excluded. The primary reasons for exclusion were (1) absence of AI model use (n=13), (2) AI not applied for diabetes prediction (n=3), (3) lack of focus on women with a history of GDM (n=4), and (4) publication types deemed irrelevant (n=5). Backward reference checking identified 1 additional eligible study. In total, 10 studies were included in this review [39-48], of which 8 met the criteria for inclusion in the meta-analyses [39-43,45,47,48]. With regard to the degree of overlap between the primary studies, the CCA across the 3 reviews was 55.9%, indicating a high degree of overlap. However, this value was largely driven by complete overlap between the 2 previous reviews, which included identical sets of primary studies (CCA=100%). Thus, we computed pairwise CCA to provide a more detailed assessment of overlap. Pairwise comparisons showed that the overlap between this review and each of the previous reviews was 35.3%, indicating moderate overlap. Notably, our review identified 4 additional and more recent studies [40,44,47,48] that were not included in the previous reviews.

### Characteristics of Included Studies

As displayed in Table 1, the reviewed studies span from 2011 to 2025. Research was conducted across 7 countries, predominantly in the United States (3/10, 30%). Most publications were journal articles (9/10, 90%). In terms of design, retrospective studies (6/10, 60%) were slightly more common than prospective studies (4/10, 40%). Reported follow-up durations varied widely, with a mean of 76.9 (SD 46.36) months and a range of 3.25 to 120 months. The included studies and their characteristics are summarized in Table 1, with additional details provided in Multimedia Appendix 5.

**Table 1.** Characteristics of the included studies

Features	Studies	References
Year of publication, n (%)		
2011	1 (10)	[46]
2016	1 (10)	[39]
2019	2 (20)	[43,44]
2020	1 (10)	[45]
2021	1 (10)	[42]
2022	1 (10)	[41]
2023	1 (10)	[47]
2025	2 (20)	[40,48]
Country of publication, n (%)		
United States	3 (30)	[39,43,45]
United Kingdom	2 (20)	[47,48]
Australia	1 (10)	[42]
Austria	1 (10)	[41]
India	1 (10)	[44]
Sweden	1 (10)	[40]
Taiwan	1 (10)	[46]
Publication type, n (%)		
Conference paper	1 (10)	[39,44]
Journal article	9 (90)	[39-43,45-48]
Study type, n (%)		
Retrospective	6 (60)	[40,41,43,45,47]
Prospective	4 (40)	[39,42,44,46,48]
Follow-up duration (months)		
Mean (SD)	76.9 (46.36)	[39-45,47]
Range	3.25‐120	[40-45,47]
Not reported, n (%)	2 (20)	[46,48]

### Characteristics of Participants

Participant characteristics are presented in Table 2, which provides detailed information on sample size, demographics, and diagnostic criteria. None of the included studies used a dataset from open sources (Table 2). The studies included an average of 804.9 (SD 1834; range 75‐6000) participants. On average, studies recruited 68.6 (SD 53.49; range 17‐173) cases of T2DM and 159 (SD 142.7; range 58‐485) controls. The mean age of participants was 33.2 (SD 0.96; range 31.9‐34.5) years, and the mean BMI was 29.4 (SD 3.19; range 25.9‐33.2) kg/m^2^. Regarding diagnostic criteria, the oral glucose tolerance test was the most frequently used reference standard (7/10, 70%). The most common guideline used to diagnose T2DM was the ADA criteria (4/10, 40%). Multimedia Appendix 6 presents the characteristics of participants in each study.

**Table 2.** Characteristics of participants in the included studies.

Characteristics	Studies	References
Data source, n (%)		
Closed	10 (100)	[39-48]
Number of participants		
Mean (SD)	804.9 (1834)	[39-45,47,48]
Range	75‐6000	[39-48]
Number of cases		
Mean (SD)	68.6 (53.49)	[39-47]
Range	17‐173	[39-47]
Not reported, n (%)	1 (10)	[48]
Number of controls		
Mean (SD)	159 (142.7)	[39-47]
Range	58‐485	[39-47]
Not reported, n (%)	1 (10)	[48]
Mean age		
Mean (SD)	33.2 (0.96)	[39-43,45,47]
Range	31.9‐34.5	[39-43,45,47]
Not reported, n (%)	3 (30)	[44,46,48]
Mean BMI		
Mean (SD)	29.4 (3.19)	[39-43,45,47]
Range	25.9‐33.2	[39-43,45,47]
Not reported, n (%)	3 (30)	[44,46,48]
Reference standard, n (%)		
Oral OGTT^a^	7 (70)	[39,40,42,43,45-47]
EHR^b^	2 (20)	[39,45]
HbA_1c_^c^	2 (20)	[40,47]
Self-reported	2 (20)	[40,44]
IM-IVGTT^d^	1 (10)	[41]
FPG^e^	1 (10) [40]	—
Not reported	1 (10)	[48]
Guidelines used, n (%)		
ADA^f^	4 (40)	[39,42,45,47]
WHO^g^	1 (10)	[40]
National guidelines	1 (10)	[46]
Not reported	4 (40)	[41,43,44,48]

a OGTT: oral glucose tolerance test.

b EHRs: electronic health record.

c HbA_1C_: Hemoglobin A_1c_.

d IM-IVGTT: insulin-modified intravenous glucose tolerance test.

e FPG: fasting plasma glucose.

f ADA: American Diabetes Association.

g WHO: World Health Organization.

### Characteristics of AI Models

The distribution and characteristics of AI models used across studies are summarized in Table 3. The most frequently used algorithms in the included studies were DT and LogReg, each used in 70% (7/10) of studies, followed by RF (5/10, 50%) and NB (4/10, 40%). The best-performing models in the studies were LogReg and RF, each achieving top performance in 30% (3/10) of studies. Most studies (9/10, 90%) applied AI models to predict T2DM, while only 3 studies used AI models to predict prediabetes. The average number of features used to develop AI models in the included studies was 128.6 (SD 225.8), ranging from 10 to 626 features, covering laboratory features in 10 of 10 studies, anthropometric features in 6 of 10 studies, omics features in 6 of 10 studies, demographic features in 6 of 10 studies, and clinical features in 5 of 10 studies. The most common features used in the included studies were fasting plasma glucose (FPG; 8/10, 80%), age (6/10, 60%), BMI (6/10, 60%), weight (5/10, 50%), and 2-hour plasma glucose (5/10, 50%). Among significant predictors, FPG and BMI were most recurrent (4/10, 40% each). The most common validation approaches used in the included studies were K-fold cross-validation (5/10, 50%) and hold-out methods (4/10, 40%). Model performance was commonly evaluated through sensitivity (8/10, 80%), accuracy (7/10, 70%), AUC (7/10, 70%), and *F*_1_-score (7/10, 70%). The characteristics of AI models in each included study are described in Multimedia Appendix 7.

**Table 3.** Characteristics of AI^a^ models.

Feature	Studies	References
AI models used, n (%)		
Decision tree	7 (70)	[39,41,43,44,46-48]
Logistic regression	7 (70)	[39-42,46-48]
Random forest	5 (50)	[40,44,45,47,48]
Naïve Bayes	4 (40)	[39,41,44,48]
Gradient boosting	3 (30)	[40,47,48]
Support vector machine	3 (30)	[44,46,48]
AdaBoost	2 (20)	[44,48]
Bagging classifier	2 (20)	[47,48]
CatBoost	2 (20)	[47,48]
Light gradient boosting machine	2 (20)	[47,48]
Extreme gradient boosting	2 (20)	[47,48]
Others	1 (10)	[46,48]
Best performing AI model, n (%)		
Logistic regression	3 (30)	[41,42,47]
Random forest	3 (30)	[40,44,45]
Decision tree	2 (20)	[39,43]
Naïve Bayes	1 (10)	[44]
AIRS^b^	1 (10)	[46]
AdaBoost	1 (10)	[48]
Target condition, n (%)		
Type 2 diabetes	9 (90)	[39-46,48]
Prediabetes	3 (30)	[40,46,47]
Number of features used		
Mean (SD)	128.6 (225.8)	[39-48]
Range	10‐626	[39-48]
Category of features, n (%)		
Laboratory	10 (100)	[39-48]
Anthropometric	6 (60)	[40-42,46-48]
Omics	6 (60)	[39,40,42,43,45,48]
Demographic	6 (60)	[40-42,46-48]
Clinical	5 (50)	[40,43,46-48]
Features used, n (%)		
Fasting plasma glucose	8 (80)	[39,41-43,45-48]
Age	6 (60)	[40-42,46-48]
BMI	6 (60)	[40-42,46-48]
Weight	5 (50)	[40,41,46-48]
2-hour plasma glucose	5 (50)	[39,43,45,46,48]
Family history of DM^c^	3 (30)	[43,46,48]
Cholesterol	2 (20)	[40,42]
Hexose	2 (20)	[39,45]
HbA_1c_^d^	2 (20)	[47,48]
Type of GDM^e^ treatment	2 (20)	[43,48]
Parity	2 (20)	[47,48]
Smoking status	2 (20)	[47,48]
Height	2 (20)	[41,47]
Mode of delivery	2 (20)	[47,48]
Blood pressure	2 (20)	[40,47]
Breastfeeding status	2 (20)	[47,48]
Others	1 (10)	[39-48]
Significant features, n (%)		
Fasting plasma glucose	4 (40)	[39,41,42,47]
BMI	4 (40)	[40-42,48]
Age	2 (20)	[41,42]
Hexose	2 (20)	[39,45]
Others	1 (10)	[39-48]
Type of validation, n (%)		
K-fold cross-validation	5 (50)	[40,41,43,44,46]
Hold-out	4 (40)	[39,42,45,48]
Nested cross-validation	1 (10)	[47]
Model performance metric, n (%)		
Sensitivity	8 (80)	[39,41-46,48]
Accuracy	7 (70)	[39,41,43-45,47,48]
Area under the curve	7 (70)	[39-43,45,47]
*F*_1_-score	7 (70)	[39,41,43-45,47,48]
Specificity	6 (60)	[39,41-45]
Precision	5 (50)	[41,43,45,47,48]
Negative predictive value	1 (10)	[47]

a AI: artificial intelligence.

b AIRS: Artificial Immune Recognition System.

c DM: diabetes mellitus.

d HbA_1c_: hemoglobin A_1c_.

e GDM: gestational diabetes mellitus.

### Results of Risk of Bias Appraisal

**Figure 2.**
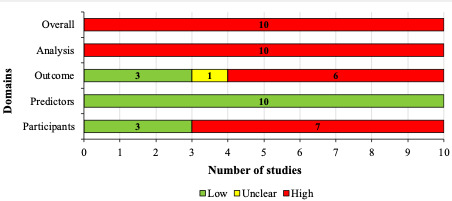
Results of assessment of risk of bias in the included studies using the PROBAST+AI tool across four domains (participants, predictors, outcome, and analysis) and overall rating. The figure shows the number of studies classified as low (green), unclear (yellow), or high (red) risk of bias in each domain for studies evaluating artificial intelligence–based models predicting progression from gestational diabetes mellitus to type 2 diabetes mellitus or prediabetes. All studies were rated as high risk of bias overall, primarily driven by limitations in the analysis domain, while the predictors domain consistently demonstrated low risk across all studies.

As previously noted, the PROBAST+AI bias assessment tool [21] assesses risk across 4 domains—participants, predictors, outcome, and analysis. In the participants domain, all 10 of 10 studies (100%) used appropriate data sources, demonstrating strong data reliability and representativeness. However, only 40% of studies (4/10) adopted appropriate study designs, and 50% (5/10) applied suitable inclusion and exclusion criteria. Therefore, 70% (7/10) of studies were judged as high risk of bias in the participants domain, while 30% (3/10) showed low risk of bias in this domain. The detailed results of the risk of bias assessment are presented in Figure 2.

In the predictors domain, all studies (10/10, 100%) clearly defined and assessed predictors in a similar way for all participants and implemented consistent preprocessing procedures. Each study also confirmed that predictor assessment was independent of outcome knowledge and that all predictors were available at the time of intended model use. Consequently, this domain reflected the highest methodological robustness, with all studies rated as low risk of bias (Figure 2).

For the outcome domain, 70% of studies (7/10) appropriately defined and assessed outcomes, and only half (5/10, 50%) determined outcomes consistently across participants. In contrast, 90% (9/10) ensured that outcome assessment was performed without previous knowledge of predictor data. A small proportion of studies (1/10, 10%) presented insufficient information regarding outcome measurement timing or standardization. Accordingly, this domain displayed mixed quality (6/10, 60% of studies were judged low risk; 3/10, 30% high risk; and 1/10, 10% unclear; Figure 2).

Finally, for the analysis domain, the most variability was visible. All studies (10/10, 100%) avoided evaluation based solely on apparent model performance. However, 90% (9/10) did not justify an adequate sample size, and 1 study (1/10, 10%) lacked sufficient data for this criterion. Most studies (9/10, 90%) properly handled missing data and addressed class imbalance. Still, occasional issues in analytical transparency and reporting were observed. Thus, the analysis domain was rated as high risk in all studies (Figure 2).

**Figure 3.**
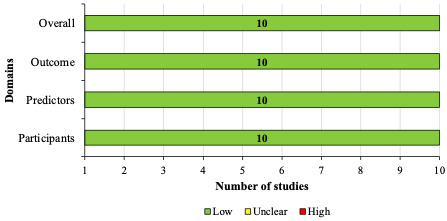
Results of assessment of applicability concerns in the included studies using the PROBAST+AI tool across domains (participants, predictors, and outcome) and overall rating. The figure presents the number of studies classified as low (green), unclear (yellow), or high (red) concern regarding applicability for studies evaluating artificial intelligence–based models predicting progression from gestational diabetes mellitus to type 2 diabetes mellitus or prediabetes. All included studies were judged to have low applicability concerns across all domains, indicating strong alignment between study populations, predictors, outcomes, and the objectives of this review.

As illustrated in Figure 2, the overall domain was rated as high risk of bias in all studies, given that each study was rated as high risk of bias in at least 1 domain. With regard to applicability concerns, all included studies (n=10/10, 100%) were judged to have a low concern across all domains because the study participants, predictors, and outcomes were well-aligned with the review’s objectives and target setting. Applicability concerns across studies are illustrated in Figure 3. A detailed breakdown of the “risk of bias” and “applicability concerns” for each domain in every study is available in Multimedia Appendix 8.

### Results of the Studies

As mentioned earlier, 8 studies provided sufficient data to be included in the meta-analyses [39-43,45,47,48]. Meta-analyses were carried out to pool results related to 2 target conditions—T2DM and prediabetes. The following subsections present the pooled results for each target condition.

### Accuracy

We conducted a meta-analysis of the highest accuracy estimates for the best-performing AI model in predicting T2DM in each study, derived from 1683 participants across 6 of 10 studies (Table 4 and Multimedia Appendix 9). The pooled accuracy of these best-model estimates was 0.85 (95% CI 0.79-0.90). The meta-analyzed evidence exhibited considerable statistical heterogeneity (τ^2^=0.007; *I*²=79%; *P*<.001). In a meta-analysis of the highest accuracy estimates for the worst-performing AI model in each study, the pooled accuracy was 0.79 (95% CI 0.70-0.87), with considerable heterogeneity (τ^2^=0.016; *I*²=92%; *P*<.001). Subgroup analyses showed that the pooled accuracy was comparable across AI models, ranging from 0.79 (95% CI 0.65-0.90) for NB to 0.84 (95% CI 0.76-0.92) for RF, with high levels of heterogeneity across studies for all models (Figures S1-S3 in Multimedia Appendix 10). No meta-analysis was conducted for predicting prediabetes, as data on accuracy were available for just 1 of 10 studies.

**Table 4.** Pooled mean estimates of accuracy in predicting type 2 diabetes mellitus. Not all studies reported all performance metrics. Missing values reflect metrics that were not reported by the original study. Differences in reported metrics may limit direct comparability across studies.

Groups	Studies, n	Sample size, n	Accuracy (%), range	Pooled mean accuracy (%), mean (95% CI)	Heterogeneity measures			
					Tau^2^	*P* value	*I*² (%)	Prediction interval
Algorithm								
Decision tree	4	1499	0.71‐0.91	0.83 (0.75 to 0.9)	.008	.006	78	0.53‐0.99
Logistic regression	4	1441	0.64‐0.89	0.83 (0.71 to 0.92)	.017	.007	90	0.38‐1.00
Naïve Bayes	3	1359	0.66‐0.87	0.79 (0.65 to 0.9)	.016	.008	93	0.19‐1.00
Random forest	2	1302	0.79‐0.88	0.84 (0.76 to 0.92)	.005	.03	79	—^a^
Overall best models	6	1683	0.71‐0.91	0.85 (0.79 to 0.9)	.007	<.001	79	0.64‐0.98
Overall worst models	6	1683	0.64‐0.91	0.79 (0.7 to 0.87)	.016	<.001	92	0.46‐0.99

a Not applicable.

### Sensitivity

We conducted a meta-analysis of the highest sensitivity estimates for the best-performing AI model in predicting T2DM in each study, which were derived from 466 participants across 5 of 10 studies (Table 5 and Multimedia Appendix 11). The pooled sensitivity of these best-model estimates was 0.89 (95% CI 0.81-0.95). The meta-analyzed evidence exhibited considerable statistical heterogeneity (τ^2^=0.009; *I*^2^=77%; *P*<.01). In a meta-analysis of the highest sensitivity estimates for the worst-performing AI model in each study, the pooled sensitivity was 0.76 (95% CI 0.63-0.86), with considerable heterogeneity (τ^2^=0.017; *I*²=76%; *P*<.01). Subgroup analyses demonstrated that the pooled sensitivity was comparable across AI models, ranging from 0.73 (95% CI 0.38-0.97) for NB to 0.81 for DT (95% CI 0.68-0.92) and RF (95% CI 0.77-0.85), with high levels of heterogeneity across studies for all models except for RF, for which no heterogeneity was detected (Figures S4-S6 in Multimedia Appendix 10). No meta-analysis was conducted for predicting prediabetes, as no usable data on sensitivity were reported by the included studies.

**Table 5.** Pooled mean estimates of sensitivity in predicting type 2 diabetes mellitus. Not all studies reported all performance metrics. Missing values reflect metrics that were not reported by the original study. Differences in reported metrics may limit direct comparability across studies.

Groups	Studies, n	Sample size, n	Sensitivity (%), range	Pooled mean sensitivity (%), mean (95% CI)	Heterogeneity measures			
					Tau^2^	*P* value	*I*² (%)	Prediction interval
Algorithm								
Decision tree	3	404	0.70‐0.88	0.81 (0.68 to 0.92)	.014	.006	85	0.23‐1.00
Logistic regression	3	360	0.57‐0.91	0.80 (0.56 to 0.97)	.041	.006	91	0.00‐1.00
Naïve Bayes	2	349	0.55‐0.87	0.73 (0.38 to 0.97)	.063	.006	95	—^a^
Random forest	2	358	0.78‐0.81	0.81 (0.77 to 0.85)	.000	.61	0	—
Overall best models	5	466	0.78‐0.95	0.89 (0.81 to 0.95)	.009	.007	77	0.63‐1.00
Overall worst models	5	466	0.55‐0.91	0.76 (0.63 to 0.86)	.017	.005	76	0.36‐1.00

a Not applicable.

### Specificity

We conducted a meta-analysis of the highest specificity estimates for the best-performing AI model in predicting T2DM in each study, which were derived from 1142 participants across 5 of 10 studies (Table 6 and Multimedia Appendix 12). The pooled specificity of the performing models of these estimates was 0.88 (95% CI 0.81-0.93). The meta-analyzed evidence exhibited moderate levels of heterogeneity (τ^2^=0.006; *I*^2^=67%; *P*=.02). In a meta-analysis of the highest specificity estimates for the worst-performing AI model in each study, the pooled specificity was 0.79 (95% CI 0.65-0.90), with considerable heterogeneity (τ^2^=0.029; *I*²=96%; *P*<.01). Subgroup analyses showed that the pooled specificity was comparable across AI models, ranging from 0.84 (95% CI 0.72-0.93) for NB to 0.87 (95% CI 0.76-0.94) for RF, with moderate to high levels of heterogeneity across studies for all models (Figures S7-S9 in Multimedia Appendix 10). No meta-analysis was conducted for predicting prediabetes, as no usable data on specificity were reported by the included studies.

**Table 6.** Pooled mean estimates of specificity in predicting type 2 diabetes mellitus. Not all studies reported all performance metrics. Missing values reflect metrics that were not reported by the original study. Differences in reported metrics may limit direct comparability across studies.

Groups	Studies, n	Sample size, n	Specificity (%), range	Pooled mean specificity (%), mean (95% CI)	Heterogeneity measures			
					Tau^2^	*P* value	*I*² (%)	Prediction interval
Algorithm								
Decision tree	3	1020	0.69‐0.93	0.85 (0.71 to 0.95)	.020	.008	83	0.18‐1.00
Logistic regression	3	1006	0.71‐0.89	0.85 (0.74 to 0.93)	.011	.02	75	0.35‐1.00
Naïve Bayes	2	935	0.76‐0.87	0.84 (0.72 to 0.93)	.008	.06	72	—^a^
Random forest	2	944	0.8‐0.9	0.87 (0.76 to 0.94)	.007	.05	73	—
Overall best models	5	1142	0.76‐0.93	0.88 (0.81 to 0.93)	.006	.02	67	0.67‐0.99
Overall worst models	5	1142	0.58‐0.93	0.79 (0.65 to 0.9)	.029	.005	96	0.29‐1.00

a Not applicable.

### *F*_1_-Score

We conducted a meta-analysis of the highest *F*_1_-score estimates for the best-performing AI model in predicting T2DM in each study, which were derived from 1608 participants across 5 of 10 studies (Table 7 and Multimedia Appendix 13). The pooled *F*_1_-score of these estimates was 0.80 (95% CI 0.75-0.85). The meta-analyzed evidence exhibited moderate statistical heterogeneity (τ^2^=0.001; *I*^2^=42%; *P*=.14). In a meta-analysis of the highest *F*_1_-score estimates for the worst-performing AI model in each study, the pooled *F*_1_-score was 0.72 (95% CI 0.60-0.84), with considerable heterogeneity (τ^2^=0.014; *I*²=91%; *P*<.01). Subgroup analyses indicated that the pooled *F*_1_-score was comparable across AI models, ranging from 0.71 (95% CI 0.56-0.86) for NB to 0.77 (95% CI 0.74-0.81) for RF, with high levels of heterogeneity across studies for all models except for RF, for which no heterogeneity was detected (Figures S10-S12 in Multimedia Appendix 10). No meta-analysis was conducted for predicting prediabetes, as no usable data on *F*_1_-score were reported by the included studies.

**Table 7.** Pooled mean estimates of *F*_1_-score in predicting type 2 diabetes mellitus. Not all studies reported all performance metrics. Missing values reflect metrics that were not reported by the original study. Differences in reported metrics may limit direct comparability across studies.

Groups	Studies, n	Sample size, n	Specificity (%), range	Pooled mean specificity (%), mean (95% CI)	Heterogeneity measures			
					Tau^2^	*P* value	*I*² (%)	Prediction interval
Algorithm								
Decision tree	3	1424	0.68‐0.88	0.76 (0.64 to 0.89)	.011	.006	91	0.56‐0.78
Logistic regression	3	1366	0.62‐0.8	0.72 (0.6 to 0.85)	.008	.04	70	0.56‐0.77
Naïve Bayes	2	1284	0.61‐0.77	0.71 (0.56 to 0.86)	.010	.04	75	—^a^
Random forest	2	1302	0.77‐0.79	0.77 (0.74 to 0.81)	.000	.68	0	—
Overall best models	5	1608	0.69‐0.88	0.8 (0.75 to 0.85)	.001	.14	42	0.68‐0.93
Overall worst models	5	1608	0.60‐0.88	0.72 (0.6 to 0.84)	.014	.007	91	0.36‐1.00

a Not applicable.

### AUC

We conducted a meta-analysis of the highest AUC estimates for the best-performing AI model in predicting T2DM in each study, which were derived from 533 participants across 6 of 10 studies (Table 8 and Multimedia Appendix 14). The pooled AUC of these estimates was 0.86 (95% CI 0.77-0.91). The meta-analyzed evidence exhibited high statistical heterogeneity (τ^2^=0.310; *I*^2^=67%; *P*<.01). In a meta-analysis of the highest AUC estimates for the worst-performing AI model in each study, the pooled AUC was 0.82 (95% CI 0.70-0.90), with considerable heterogeneity (τ^2^=0.607; *I*²=81%; *P*<.01). Subgroup analyses indicated that the pooled AUC score was comparable across AI models, ranging from 0.78 (95% CI 0.69-0.85) for NB to 0.84 (95% CI 0.71-0.92) for DT, with high levels of heterogeneity across studies for all models except for NB, for which no heterogeneity was detected (Figures S13-S15 in Multimedia Appendix 10).

**Table 8.** Pooled mean estimates of area under the curve score in predicting type 2 diabetes mellitus. Not all studies reported all performance metrics. Missing values reflect metrics that were not reported by the original study. Differences in reported metrics may limit direct comparability across studies.

Groups	Studies, n	Sample size, n	Specificity (%), range	Pooled mean specificity (%), mean (95% CI)	Heterogeneity measures			
					Tau^2^	*P* value	*I*² (%)	Prediction interval
Algorithm								
Decision tree	3	299	0.77‐0.92	0.84 (0.71 to 0.92)	.352	.01	76	0.20‐0.99
Logistic regression	4	291	0.56‐0.92	0.79 (0.59 to 0.91)	.745	.007	77	0.14‐0.99
Naïve Bayes	2	159	0.75‐0.83	0.78 (0.69 to 0.85)	.000	.34	0	—^a^
Random forest	2	152	0.67‐0.88	0.8 (0.53 to 0.94)	.705	.02	82	—
Overall best models	6	533	0.67‐0.92	0.86 (0.77 to 0.91)	.310	.008	67	0.54‐0.97
Overall worst models	6	533	0.56‐0.92	0.82 (0.7 to 0.9)	.607	.007	81	0.34‐0.98

a Not applicable.

We conducted a meta-analysis of the highest AUC estimates for the best-performing AI model in predicting prediabetes in each study, which were derived from 454 participants across 2 of 10 studies (Table 9 and Multimedia Appendix 14). The pooled AUC of these estimates was 0.69 (95% CI 0.60-0.77). The meta-analyzed evidence exhibited moderate statistical heterogeneity (τ^2^=0.038; *I*^2^=37%; *P*=.21). In a meta-analysis of the highest AUC estimates for the worst-performing AI model in each study, the pooled AUC was 0.61 (95% CI 0.55-0.67), with low heterogeneity (τ^2^=0.00; *I*²=37%; *P*=.48). Subgroup analyses indicated that the pooled AUC score was comparable across AI models, ranging from 0.66 (95% CI 0.69-0.85) for LogReg to 0.68 for RF and XGBoost, with no to low heterogeneity across studies for all models except for LogReg, for which the heterogeneity level was high (Figures S16-S18 in Multimedia Appendix 10). AUC was substantially lower for prediabetes than for T2DM (0.69 vs 0.86).

**Table 9.** Pooled mean estimates of area under the curve score in predicting prediabetes. Not all studies reported all performance metrics. Missing values reflect metrics that were not reported by the original study. Differences in reported metrics may limit direct comparability across studies.

Groups	Studies, n	Sample size, n	Specificity (%), range	Pooled mean specificity (%), mean (95% CI)	Heterogeneity measures			
					Tau^2^	*P* value	I² (%)	Prediction interval
Algorithm								
Random forest	2	454	0.62‐0.69	0.68 (0.62-0.74)	.000	.36	0	—^a^
Gradient boosting	2	454	0.61‐0.7	0.68 (0.61-0.75)	.013	.27	17	—
Logistic regression	2	454	0.56‐0.72	0.66 (0.49-0.79)	.184	.05	74	—
Overall best models	2	454	0.62‐0.72	0.69 (0.6-0.77)	.038	.21	37	—
Overall worse models	2	454	0.56‐0.65	0.61 (0.55-0.67)	.000	.48	0	—

a Not applicable.

### Publication Bias

Publication bias was evaluated using funnel plot inspection and Egger regression test. The funnel plots for accuracy, sensitivity, specificity, *F*_1_-score, and AUC appeared largely symmetrical, with no clear evidence of small-study effects (Multimedia Appendix 15). Correspondingly, the Egger test indicated no statistically significant funnel plot asymmetry for any performance metric (accuracy: *P*=.18; sensitivity: *P*=.12; specificity: *P*=.18; *F*_1_-score: *P*=.59; and AUC: *P*=.75). Collectively, these findings suggest that the pooled estimates are unlikely to be meaningfully influenced by publication bias. However, it is important to interpret these findings cautiously. Funnel plot asymmetry tests, including Egger regression, have limited statistical power when fewer than 10 studies are included in a meta-analysis. Given the small number of studies contributing to each pooled estimate, the absence of statistically significant asymmetry does not definitively exclude the possibility of publication bias or small-study effects. Therefore, conclusions regarding publication bias should be considered provisional.

## Discussion

### Principal Findings

This systematic review and meta-analysis aimed to evaluate the performance of AI models in predicting the progression from GDM to T2DM. Overall, the findings indicate that AI models show strong capability in predicting progression to T2DM, whereas their performance is more limited for identifying prediabetes, suggesting greater difficulty in detecting early-stage dysglycemia, indicating that early subclinical dysglycemia is more challenging to detect, possibly due to limited biomarker resolution or small prediabetes datasets. Across studies, different AI algorithms showed comparable performance, suggesting that predictive accuracy is largely driven by the underlying data rather than the choice of algorithm [49,50]. Additionally, FPG and BMI consistently emerged as the most important predictors of progression.

The included studies exhibited substantial methodological and clinical heterogeneity, which likely contributed to the high statistical heterogeneity observed across most pooled analyses (*I*² frequently exceeding 70%). Considerable variability existed in follow-up duration, study design, participant characteristics, predictor sets, and model validation strategies. For instance, follow-up periods ranged from approximately 3 to 120 months, potentially influencing both the observed incidence of T2DM and the temporal stability of predictive features. Models developed using shorter follow-up intervals may primarily capture early metabolic deterioration, whereas studies with longer follow-up durations may reflect broader long-term risk trajectories. Additionally, the number and type of predictors varied widely across studies, ranging from basic clinical and anthropometric variables to multiomics data, which may influence both model complexity and predictive performance. Differences in validation strategies may further contribute to variability in reported performance estimates, as most studies relied on internal validation methods (eg, cross-validation or hold-out testing), which are known to produce more optimistic estimates than independent external validation [51].

Given this variability, the pooled estimates derived from the meta-analyses should be interpreted with caution, as the underlying evidence is characterized by substantial heterogeneity and high risk of bias across included studies. These methodological limitations may influence both the magnitude and variability of the pooled estimates [52]. PIs were therefore reported to provide an additional measure of uncertainty, reflecting the expected range of effect estimates in future settings beyond the average weighted estimate. Accordingly, the pooled results should be interpreted as indicative summaries of model performance across diverse methodological contexts rather than precise or universally generalizable estimates.

Moreover, across all performance metrics for T2DM and prediabetes prediction, pooled estimates from best-performing models were consistently higher than those from worst-performing models. These findings suggest that the pooled estimates derived from best-performing models likely represent upper-bound performance, whereas the worst-model analyses provide a conservative lower-bound estimate. The true performance of AI models in real-world settings likely lies between these bounds, underscoring the importance of cautious interpretation and the need for external validation [49].

An important finding of this review is that all included studies were judged to have an overall high risk of bias according to the PROBAST+AI assessment. This result has important implications for interpreting the pooled performance estimates. High risk of bias, particularly within the analysis domain, may lead to overly optimistic estimates of predictive performance [24] due to issues such as small sample sizes, inadequate reporting of model development procedures, potential overfitting, and reliance on internal validation methods. Consequently, the pooled estimates of accuracy, sensitivity, specificity, and AUC should be interpreted as indicative summaries of reported performance rather than precise estimates of real-world predictive capability. The true performance of these models in clinical practice may be lower than the values reported in the primary studies.

Our findings were different from the 2 previous reviews [15,16]. This can be attributed to the fact that both previous reviews included the same 13 studies; however, 6 of these [17-22] did not fully meet the scope of the present review for the reasons mentioned in the Research Problem and Aim section. Specifically, Zhao et al [16] included 13 studies and 23 AI models and reported a sensitivity of 0.76 and a specificity of 0.57. In contrast, after excluding studies that used non-AI methods or assessed unrelated outcomes, our review found higher pooled sensitivity and specificity for T2DM prediction, at 0.89 and 0.88, respectively. Elfadel Magboul et al [15] did not conduct a meta-analysis but reported AUC values ranging from 0.72 to 0.92, which overlaps with the range observed in our review. However, because that review included studies with different modeling approaches and outcomes, its reported performance range should be interpreted as reflecting a broader and more heterogeneous prediction literature rather than AI-specific performance for GDM-to-T2DM progression. Therefore, this review provides a more focused estimate of AI model performance by restricting inclusion to studies that directly applied AI algorithms to predict T2DM or prediabetes among women with previous GDM. The exclusion of the 6 studies [17-22] does not imply that those studies lack scientific value. Rather, they address different research questions or use different modeling traditions. Conventional clinical risk scores and regression-based models can be useful for risk stratification, and models predicting postpartum screening attendance may help improve follow-up care. However, combining these studies with AI-based prediction models of diabetes progression can obscure the specific contribution of AI and may affect pooled estimates, particularly when studies differ in outcome definition, algorithmic approach, validation strategy, and performance metrics. By applying stricter eligibility criteria, this review clarifies the evidence specifically attributable to AI models and provides a more clinically coherent synthesis for postpartum diabetes risk prediction.

Across all pooled metrics, the AI models (RF, DT, LogReg, and NB) exhibited comparable predictive performance, suggesting that algorithm selection may have a limited effect on overall model performance in this context. This finding is consistent with the study by Zhao et al [16], which found no significant difference in the performance of these algorithms. This convergence in performance likely reflects several underlying factors. First, the datasets used across studies were moderate in size, low in dimensionality, and dominated by well-established clinical predictors (eg, fasting glucose, BMI, age, and family history of diabetes). Such structured and relatively low-noise data are typically well-modeled by both linear and nonlinear approaches, diminishing the performance gap between algorithmic families [53-55]. In these scenarios, simpler algorithms such as LogReg can perform nearly as well as more complex ensemble or tree-based methods because the underlying relationships between predictors and outcomes are largely monotonic and interpretable [50,53]. Second, comparable performance may also stem from limited feature diversity and overlap in model training variables, leading to convergent predictive boundaries [50]. When the same small set of physiologic and metabolic variables drives model performance, even fundamentally different algorithms tend to approximate similar decision surfaces [50]. Furthermore, all included studies used internal cross-validation rather than independent external datasets, which can mask true performance differences between models [56].

The most significant predictors identified across studies were FPG and BMI. These findings are consistent with Elfadel Magboul et al [15], who found that FPG and BMI were among the most influential features across multiple AI models. This reinforces their pivotal role in the pathophysiological transition from GDM to T2DM. Elevated FPG levels during or after pregnancy reflect persistent insulin resistance and β-cell dysfunction, which is a core mechanism driving the eventual progression to overt diabetes [57,58]. Even modest elevations in fasting glucose postpartum indicate incomplete metabolic recovery following GDM and may signify diminished β-cell reserve [59]. Similarly, higher BMI serves as a robust marker of increased adiposity, which promotes chronic low-grade inflammation, dysregulated lipid metabolism, and impaired insulin signaling [60,61]. The coexistence of residual hyperglycemia and elevated BMI thus represents a synergistic metabolic burden that accelerates the deterioration of glucose tolerance over time [62,63].

An important methodological contribution of this review is the formal assessment of overlap between included studies and previous systematic reviews using the CCA. The high overall CCA (55.9%) suggests substantial redundancy in the existing evidence base, largely driven by complete overlap between earlier reviews. However, the moderate pairwise overlap (35.3%) between this review and previous reviews indicates that this study contributes additional and more recent evidence. This highlights both the rapid evolution of the field and the importance of regularly updating systematic reviews to capture newly published studies.

The risk of bias findings in this review differ from those reported in the 2 previous systematic reviews [15,16]. This difference can be attributed to two reasons: (1) the overlap between our review and the previous 2 reviews in terms of the included studies is only 35.3%, and (2) we used the AI-specific PROBAST+AI tool [24] rather than the original PROBAST tool [23]. To be more specific, Elfadel Magboul et al [15] assessed 13 studies using PROBAST [23] and concluded that most studies had a low risk of bias across the participants, predictors, outcome, and analysis domains, although several studies had unclear or high risk mainly due to insufficient validation details and analytical limitations. In contrast, Zhao et al [16] also used PROBAST [23], identified more methodological concerns, including high risk in the predictor domain for several models, unclear risk in the outcome domain for some models, and high risk in the analysis domain for models that lacked adequate events per variable or independent validation. In our review, all included studies were judged to have a high overall risk of bias, primarily because every study had a high risk in at least 1 domain. Specifically, 70% (7/10) of studies were rated as high risk in the participants domain, all studies were rated as low risk in the predictors domain, 60% (6/10) were low risk, 30% (3/10) were high risk, and 10% (1/10) were unclear risk in the outcome domain, and all studies were rated as high risk in the analysis domain.

The use of PROBAST+AI therefore contributes new knowledge beyond the previous reviews by distinguishing between clinical relevance and AI methodological reliability. While the previous PROBAST-based reviews suggested that many models were methodologically acceptable, this PROBAST+AI assessment shows that the evidence base remains at high risk of bias when AI-specific issues are considered [24]. These include inadequate sample size relative to model complexity, insufficient or unclear validation, possible overfitting, limited external validation, handling of class imbalance, preprocessing consistency, and transparency in model development and evaluation [24]. This is particularly important because high-pooled performance estimates may otherwise be interpreted as evidence of clinical readiness.

### Research and Practical Implications

Although this review showed promising results, conclusions regarding AI effectiveness should be interpreted with caution and regarded as hypothesis-generating pending large-scale, externally validated studies for several reasons. First, most of the studies included in this review were small (median 179, IQR 103-357 participants), with a median prevalence of roughly 30%, and possibly included more features than they were powered to include (median 26, IQR 15-38 features per model, roughly 1 feature per every 2 cases) [64]. Second, the number of studies included in the meta-analyses was small (2‐4 studies). Third, there was a high risk of bias in most domains of the included studies. The consistent presence of a high risk of bias substantially reduces the certainty of the evidence and limits confidence in the reported performance estimates. Fourth, none of the included studies conducted independent external validation using geographically, temporally, or institutionally distinct cohorts. All reported performance metrics were derived from internal validation strategies. While these methods are useful for preliminary evaluation, they do not adequately assess transportability across different populations or health care settings [65]. Consequently, reported discrimination metrics may reflect optimistic bias inherent to internal validation, limiting confidence in real-world applicability [66]. Finally, substantial statistical heterogeneity was observed across most pooled analyses. This heterogeneity likely reflects genuine methodological and clinical diversity rather than random variation alone [52]. Included studies differed in study design (retrospective vs prospective), follow-up duration (ranging from 3.25 to 120 months), outcome definitions (T2DM vs prediabetes), diagnostic criteria (ADA, World Health Organization, and national guidelines), and reference standards (oral glucose tolerance test, hemoglobin A_1c_, electronic health records–based diagnoses). Such heterogeneity limits the interpretability of pooled discrimination metrics and suggests that summary estimates should be viewed as indicative rather than definitive measures of performance [52]. Accordingly, there is an urgent need to conduct more studies with a large sample size. Adoption of AI-specific reporting guidelines, such as TRIPOD-AI [67] and PROBAST+AI [24], can improve reproducibility, enable meta-analytic synthesis, and reduce the risk of bias. Future research should also emphasize external and prospective validation using ethnically diverse, multicenter cohorts to ensure generalizability across populations.

From a clinical perspective, the reported performance metrics provide insight into how these models may be applied in practice. The relatively high sensitivity observed across studies suggests that AI models are well-suited for identifying women at high risk of progressing to T2DM, minimizing the likelihood of missed cases and supporting early intervention. At the same time, the high specificity indicates that these models can also be effective in ruling out low-risk individuals, potentially reducing unnecessary follow-up testing and health care burden. The balance between sensitivity and specificity suggests that these models could be deployed as risk stratification tools within postpartum care pathways, where thresholds can be adjusted depending on the clinical objective: prioritizing sensitivity when the goal is early detection, or specificity when aiming to optimize resource allocation. This flexibility enhances their potential utility in real-world settings.

Although the included studies incorporated a broad range of demographic, clinical, and laboratory features, several important predictors of T2DM progression were notably absent from the model development process. Specifically, none of the studies integrated socioeconomic (eg, education level and income), psychosocial (eg, stress level, postpartum depression, and social support), or lifestyle factors (eg, sleep quality and duration, physical activity levels, and dietary composition) despite their well-documented influence on postpartum metabolic recovery and long-term glycemic control [68,69]. Hormonal and reproductive biomarkers (eg, prolactin, cortisol, estrogen, progesterone, and sex hormone–binding globulin) were also not used, even though they may mediate insulin sensitivity and β-cell function after pregnancy [70,71]. Moreover, gut microbiome composition, inflammatory markers (eg, IL-6 and TNF-α), and other metabolic syndromes (eg, polycystic ovary syndrome or nonalcoholic fatty liver disease) were not considered by the included studies [72,73]. Additionally, from a genetic and epigenetic standpoint, only 1 study included limited T2DM-associated variants (eg, TCF7L2 and FTO), leaving out other risk alleles and DNA methylation patterns known to contribute to interindividual susceptibility [74]. Health care system variables (eg, postpartum follow-up adherence, access to diabetes education, and postpartum medication exposure [eg, metformin or insulin use]) were also excluded, despite their potential to refine risk stratification in real-world contexts [75,76]. All models in the included studies relied on single-point measurements rather than repeated measures (ie, longitudinal data) such as continuous glucose monitoring–derived metrics, postpartum weight change, and rate of weight loss or gain. Collectively, these omissions suggest that while current AI models effectively capture metabolic and anthropometric risk factors, they fail to represent the complex, multidimensional determinants of T2DM development after GDM. Incorporating these underexplored predictors into future AI frameworks, especially through multiomics, longitudinal, and real-world data integration, could markedly improve the precision and clinical applicability of predictive models.

It is important to distinguish between predictive performance observed in retrospective datasets and readiness for real-world clinical deployment. The models included in this review were predominantly developed and evaluated using retrospective data and internal validation approaches, which may not reflect performance in routine clinical settings. As such, strong predictive performance in these controlled environments does not equate to clinical readiness [77,78]. Several critical barriers must be addressed before implementation, including robust external validation across diverse populations, integration into clinical workflows and electronic health record systems, and demonstration of downstream clinical impact, such as improved screening uptake or reduced progression to T2DM. Without these steps, the translation of AI models into practice remains limited.

### Limitations

This review has several limitations that should be acknowledged. The robustness of the pooled estimates is limited due to a relatively small number of studies available for inclusion (n=10 for qualitative synthesis, n=8 for meta-analysis), significant statistical heterogeneity observed (*I*² often >75%), high risk of bias in most domains in the included studies, and lack of external validation. Furthermore, our strict inclusion criteria, while improving methodological rigor, may have excluded relevant studies, and the restriction to English-language publications could introduce a language bias.

Furthermore, due to the limited number of eligible studies, it was not possible to conduct subgroup analyses by follow-up duration, geographic region, year of publication, predictor categories, validation strategies, or participant characteristics, which may have provided further insights into sources of heterogeneity. We also excluded studies that used AI solely to predict T2DM irrespective of previous GDM diagnosis and studies that developed predictive models not based on AI algorithms. Therefore, our findings could not be generalized to other populations other than women who are diagnosed with GDM and other predictive models other than AI-based models.

Another methodological limitation relates to the pooling of diagnostic performance metrics. Sensitivity and specificity were synthesized independently rather than using hierarchical diagnostic meta-analysis models that jointly model their correlation. This decision was primarily driven by incomplete reporting of threshold-specific data and contingency tables across studies, which precluded implementation of bivariate or hierarchical summary receiver operating characteristic models. As a result, the pooled estimates represent average performance across heterogeneous AI models rather than precise threshold-specific diagnostic accuracy estimates.

PIs were estimated to reflect between-study variability; however, their interpretation should consider the high risk of bias across included studies, as methodological limitations may contribute to variability and affect the reliability of pooled estimates.

Finally, we acknowledge another limitation related to model selection. For the primary analysis, we extracted the best-performing model reported in each study to represent the model intended for potential clinical use. However, this approach may introduce optimistic bias because model performance can be influenced by internal model selection and hyperparameter tuning procedures. Although we conducted additional sensitivity analyses using worst-performing models to provide conservative estimates, future meta-analyses would benefit from standardized reporting of model development pipelines and validation strategies, including external validation.

### Conclusion

This review demonstrates that AI models may hold significant promise for predicting the progression from GDM to T2DM. Key predictors like fasting glucose and BMI consistently emerged as the strongest predictors. In contrast, model performance for prediabetes prediction was modest, indicating the need for further research to detect earlier metabolic deterioration. Despite these encouraging results, current evidence remains limited by small sample sizes, high heterogeneity, lack of external validation, and high risk of bias. All models overlook critical socioeconomic, lifestyle, and psychosocial factors, while also struggling with imbalanced datasets. Therefore, the reported performance should be considered preliminary and potentially optimistic, reflecting internally validated models under constrained study conditions rather than established real-world effectiveness.

This review offers a novel contribution by providing a comprehensive meta-analytic evaluation of AI-based models for predicting progression from GDM to T2DM, alongside the use of AI-specific risk of bias assessment (PROBAST+AI). In contrast to previous reviews that included traditional statistical models or lacked quantitative synthesis, our study delivers a methodologically rigorous and clinically relevant assessment of AI model performance. It contributes to the field by identifying key predictors such as FPG and BMI, while also highlighting important limitations in current evidence, including a lack of external validation and dataset imbalance. From a digital health perspective, these findings support the implementation of AI-driven risk stratification tools within clinical workflows and electronic health record systems, enabling more proactive screening, personalized interventions, and improved postpartum care for women with a history of GDM. For successful clinical translation, future work must prioritize large, diverse cohorts and robust external validation. Integrating multidimensional data (eg, omics, longitudinal metrics, and behavioral factors) is crucial. Additionally, future research should adopt standardized AI reporting frameworks and promote open-access data sharing. By addressing these gaps, AI can evolve into a reliable tool for personalized risk stratification, enabling early intervention and reducing the long-term burden of diabetes in this vulnerable population.

## Supplementary material

10.2196/87882 Multimedia Appendix 1 PRISMA-DTA-Checklist.

10.2196/87882 Multimedia Appendix 2 Search Strategy.

10.2196/87882 Multimedia Appendix 3 Data extraction form.

10.2196/87882 Multimedia Appendix 4 Risk of bias assessment tool (PROBAST+AI).

10.2196/87882 Multimedia Appendix 5 Characteristics of studies.

10.2196/87882 Multimedia Appendix 6 Characteristics of participants.

10.2196/87882 Multimedia Appendix 7 Characteristics of AI models.

10.2196/87882 Multimedia Appendix 8 Reviewers’ judgments about each “risk of bias” and applicability domain for each included study.

10.2196/87882 Multimedia Appendix 9 Forest plots for accuracy.

10.2196/87882 Multimedia Appendix 10 Forest plots.

10.2196/87882 Multimedia Appendix 11 Forest plots for sensitivity.

10.2196/87882 Multimedia Appendix 12 Forest plots for specificity.

10.2196/87882 Multimedia Appendix 13 Forest plots for *F*_1_-score.

10.2196/87882 Multimedia Appendix 14 Forest plots for area under the curve.

10.2196/87882 Multimedia Appendix 15 Publication bias results.

## Abbreviations

ADA: American Diabetes Association
AI: artificial intelligence
AUC: area under the curve
CCA: corrected covered area
CHARMS: Checklist for Critical Appraisal and Data Extraction for Systematic Reviews of Prediction Modelling Studies
DT: decision tree
FPG: fasting plasma glucose
GDM: gestational diabetes mellitus
LogReg: logistic regression
NB: naïve Bayes
PI: prediction interval
PRISMA-DTA: Preferred Reporting Items for Systematic Reviews and Meta-Analyses - Extension for Diagnostic Test Accuracy
PRISMA-S: Preferred Reporting Items for Systematic Reviews and Meta-Analyses literature search extension
PROBAST: Prediction Model Risk of Bias Assessment Tool
PROSPERO: International Prospective Register of Systematic Reviews
REML: restricted maximum likelihood
RF: random forest
T2DM: type 2 diabetes mellitus
XGBoost: extreme gradient boosting
