# Supplementary material for: Performance of AI in Predicting the Progression of Gestational Diabetes to Type 2 Diabetes: Systematic Review and Meta-Analysis
Source: J Med Internet Res. 2026 Jul 9;28:e87882. doi: 10.2196/87882 (PMC13349230; doi:10.2196/87882)
Supplement: Multimedia Appendix 2 [file jmir-v28-e87882-s002.docx]

**Appendix 2: Search Strategy**

Database(s): **Ovid MEDLINE(R) ALL**1946 to September 12, 2025
Search Strategy:

| **#** | **Searches** | **Results** |
| --- | --- | --- |
| 1 | exp Artificial Intelligence/ | 239238 |
| 2 | "Artificial Intelligence".tw. | 63757 |
| 3 | exp Machine Learning/ | 95466 |
| 4 | "Machine Learning".tw. | 132707 |
| 5 | exp Deep Learning/ | 30515 |
| 6 | "Deep Learning".tw. | 74878 |
| 7 | "Decision Tree*".tw. | 19267 |
| 8 | "Support Vector Machine*".tw. | 32517 |
| 9 | "Recurrent Neural Network*".tw. | 5289 |
| 10 | "Convolutional Neural Network*".tw. | 33276 |
| 11 | "Artificial Neural Network*".tw. | 20849 |
| 12 | "Deep Neural Network*".tw. | 12456 |
| 13 | "Random Forest*".tw. | 35239 |
| 14 | "Long Short-Term Memory*".tw. | 6881 |
| 15 | "Autoencoder".tw. | 3953 |
| 16 | "Gradient Boost*".tw. | 10537 |
| 17 | "Multilayer Perceptron".tw. | 3735 |
| 18 | "Ensemble learning".tw. | 2781 |
| 19 | "K-Nearest Neighbor*".tw. | 7252 |
| 20 | "Naïve Bayes".tw. | 10 |
| 21 | "Naive Bayes".tw. | 4320 |
| 22 | "AdaBoost".tw. | 2066 |
| 23 | XGboost.tw. | 7716 |
| 24 | "Predict* model*".tw. | 91949 |
| 25 | "reinforcement learning".tw. | 7726 |
| 26 | "Transfer Learning".tw. | 7403 |
| 27 | Diabetes, Gestational/ | 17950 |
| 28 | "gestational diabet*".tw. | 25316 |
| 29 | "Pregnancy-Induced diabet*".tw. | 15 |
| 30 | GDM.tw. | 13863 |
| 31 | Diabetes Mellitus, Type 2/ | 190409 |
| 32 | "type 2 diabet*".tw. | 199113 |
| 33 | "type II diabet*".tw. | 12190 |
| 34 | "Noninsulin-Dependent diabet*".tw. | 1330 |
| 35 | "Non-insulin-Dependent diabet*".tw. | 10155 |
| 36 | "Ketosis-Resistant diabet*".tw. | 14 |
| 37 | "Maturity-Onset diabet*".tw. | 2519 |
| 38 | "Adult-Onset diabet*".tw. | 591 |
| 39 | T2DM.tw. | 40837 |
| 40 | 1 or 2 or 3 or 4 or 5 or 6 or 7 or 8 or 9 or 10 or 11 or 12 or 13 or 14 or 15 or 16 or 17 or 18 or 19 or 20 or 21 or 22 or 23 or 24 or 25 or 26 | 492260 |
| 41 | 27 or 28 or 29 or 30 | 28513 |
| 42 | 31 or 32 or 33 or 34 or 35 or 36 or 37 or 38 or 39 | 269751 |
| 43 | 40 and 41 and 42 | 66 |
| 44 | limit 43 to english language | 66 |

Database(s): **Embase**1974 to 2025 Week 37
Search Strategy:

| **#** | **Searches** | **Results** |
| --- | --- | --- |
| 1 | exp Artificial Intelligence/ | 144261 |
| 2 | "Artificial Intelligence".tw. | 75339 |
| 3 | exp Machine Learning/ | 594730 |
| 4 | "Machine Learning".tw. | 154095 |
| 5 | exp Deep Learning/ | 82148 |
| 6 | "Deep Learning".tw. | 85858 |
| 7 | "Decision Tree*".tw. | 26190 |
| 8 | "Support Vector Machine*".tw. | 37968 |
| 9 | "Recurrent Neural Network*".tw. | 5885 |
| 10 | "Convolutional Neural Network*".tw. | 38401 |
| 11 | "Artificial Neural Network*".tw. | 23679 |
| 12 | "Deep Neural Network*".tw. | 13872 |
| 13 | "Random Forest*".tw. | 41800 |
| 14 | "Long Short-Term Memory*".tw. | 6842 |
| 15 | "Autoencoder".tw. | 4369 |
| 16 | "Gradient Boost*".tw. | 12188 |
| 17 | "Multilayer Perceptron".tw. | 4088 |
| 18 | "Ensemble learning".tw. | 3023 |
| 19 | "K-Nearest Neighbor*".tw. | 8282 |
| 20 | "Naïve Bayes".tw. | 27 |
| 21 | "Naive Bayes".tw. | 5238 |
| 22 | "AdaBoost".tw. | 2411 |
| 23 | XGboost.tw. | 8950 |
| 24 | "Predict* model*".tw. | 119176 |
| 25 | "reinforcement learning".tw. | 8611 |
| 26 | "Transfer Learning".tw. | 7953 |
| 27 | Diabetes, Gestational/ | 53615 |
| 28 | "gestational diabet*".tw. | 40086 |
| 29 | "Pregnancy-Induced diabet*".tw. | 25 |
| 30 | GDM.tw. | 21567 |
| 31 | Diabetes Mellitus, Type 2/ | 403576 |
| 32 | "type 2 diabet*".tw. | 314034 |
| 33 | "type II diabet*".tw. | 20706 |
| 34 | "Noninsulin-Dependent diabet*".tw. | 1618 |
| 35 | "Non-insulin-Dependent diabet*".tw. | 11911 |
| 36 | "Ketosis-Resistant diabet*".tw. | 15 |
| 37 | "Maturity-Onset diabet*".tw. | 3590 |
| 38 | "Adult-Onset diabet*".tw. | 808 |
| 39 | T2DM.tw. | 68145 |
| 40 | 1 or 2 or 3 or 4 or 5 or 6 or 7 or 8 or 9 or 10 or 11 or 12 or 13 or 14 or 15 or 16 or 17 or 18 or 19 or 20 or 21 or 22 or 23 or 24 or 25 or 26 | 799648 |
| 41 | 27 or 28 or 29 or 30 | 58910 |
| 42 | 31 or 32 or 33 or 34 or 35 or 36 or 37 or 38 or 39 | 466720 |
| 43 | 40 and 41 and 42 | 197 |
| 44 | limit 43 to english language | 197 |
| 45 | limit 44 to "remove medline records" | 97 |

| Database | Search Query | Hits |
| --- | --- | --- |
| Scopus | ( TITLE-ABS-KEY ( "Artificial Intelligence" OR "Machine Learning" OR "Deep Learning" OR "reinforcement learning" OR "Transfer Learning" OR "Decision tree" OR "K-Nearest Neighbor*" OR "Support vector machine*" OR "Recurrent neural network*" OR "convolutional neural network*" OR "Artificial neural network*" OR "Deep neural network*" OR "Naïve Bayes" OR "Naive Bayes" OR "Random Forest" OR "Long Short-Term Memory Networks" OR "Gradient Boost*" OR adaboost OR xgboost OR "Multilayer Perceptron" OR "Ensemble learning" OR "Autoencoder" OR "Predict* model*" ) AND TITLE-ABS-KEY ( "gestational diabet*" OR "Pregnancy-Induced diabet*" OR gdm ) AND TITLE-ABS-KEY ( "type 2 diabet*" OR "type II diabet*" OR "Noninsulin-Dependent diabet*" OR "Non-insulin-Dependent diabet*" OR "Ketosis-Resistant diabet*" OR "Maturity-Onset diabet*" OR "Adult-Onset diabet*" OR t2dm ) ) | 154 |
| Web of Science | "Artificial Intelligence" OR "Machine Learning" OR "Deep Learning" OR "reinforcement learning" OR "Transfer Learning" OR "Decision tree" OR "K-Nearest Neighbor*" OR "Support vector machine*" OR "Recurrent neural network*" OR "convolutional neural network*" OR "Artificial neural network*" OR "Deep neural network*" OR "Naïve Bayes" OR "Naive Bayes" OR "Random Forest" OR "Long Short-Term Memory Networks" OR "Gradient Boost*" OR AdaBoost OR XGboost OR "Multilayer Perceptron" OR "Ensemble learning" OR "Autoencoder" OR "Predict* model*" (Topic) and "gestational diabet*" OR "Pregnancy-Induced diabet*" OR GDM (Topic) and "type 2 diabet*" OR "type II diabet*" OR "Noninsulin-Dependent diabet*" OR "Non-insulin-Dependent diabet*" OR "Ketosis-Resistant diabet*" OR "Maturity-Onset diabet*" OR "Adult-Onset diabet*" OR T2DM (Topic) and Review Article (Exclude – Document Types) and English (Languages) | 62 |
| ACM Digital Library | [[All: "artificial intelligence"] OR [All: "machine learning"] OR [All: "deep learning"] OR [All: "reinforcement learning"] OR [All: "transfer learning"] OR [All: "decision tree"] OR [All: "k-nearest neighbor*"] OR [All: "support vector machine*"] OR [All: "recurrent neural network*"] OR [All: "convolutional neural network*"] OR [All: "artificial neural network*"] OR [All: "deep neural network*"] OR [All: "naïve bayes"] OR [All: "naive bayes"] OR [All: "random forest"] OR [All: "long short-term memory networks"] OR [All: "gradient boost*"] OR [All: adaboost] OR [All: xgboost] OR [All: "multilayer perceptron"] OR [All: "ensemble learning"] OR [All: "autoencoder"] OR [All: "predict* model*"]] AND [[All: "gestational diabet*"] OR [All: "pregnancy-induced diabet*"] OR [All: gdm]] AND [[All: "type 2 diabet*"] OR [All: "type ii diabet*"] OR [All: "noninsulin-dependent diabet*"] OR [All: "non-insulin-dependent diabet*"] OR [All: "ketosis-resistant diabet*"] OR [All: "maturity-onset diabet*"] OR [All: "adult-onset diabet*"] OR [All: t2dm]] | 4 |
| IEEE Xplore | ("Abstract":"Artificial Intelligence" OR "Abstract":"Machine Learning" OR "Abstract":"Deep Learning" OR "Abstract":"reinforcement learning" OR "Abstract":"Transfer Learning" OR "Abstract":"Decision tree" OR "Abstract":"K-Nearest Neighbor*" OR "Abstract":"Support vector machine*" OR "Abstract":"Recurrent neural network*" OR "Abstract":"convolutional neural network*" OR "Abstract":"Artificial neural network*" OR "Abstract":"Deep neural network*" OR "Abstract":"Naïve Bayes" OR "Abstract":"Naive Bayes" OR "Abstract":"Random Forest" OR "Abstract":"Long Short-Term Memory Networks" OR "Abstract":"Gradient Boost*" OR "Abstract":AdaBoost OR "Abstract":XGboost OR "Abstract":"Multilayer Perceptron" OR "Abstract":"Ensemble learning" OR "Abstract":"Autoencoder" OR "Abstract":"Predict* model*") AND ("Abstract":"gestational diabetes" OR "Abstract":"Pregnancy-Induced diabetes" OR "Abstract":GDM) AND ("Abstract":"type 2 diabetes" OR "Abstract":"type II diabetes" OR "Abstract":"Noninsulin-Dependent diabetes*" OR "Abstract":"Non-insulin-Dependent diabetes" OR "Abstract":"Ketosis-Resistant diabetes" OR "Abstract":"Maturity-Onset diabetes" OR "Abstract":"Adult-Onset diabetes" OR "Abstract":T2DM) | 12 |
| Google Scholar | ("gestational diabet*" OR GDM) AND ("type 2 diabet*" OR "type II diabet*" OR T2DM) AND ("Artificial Intelligence" OR "Machine Learning" OR "Deep Learning" OR "Decision tree" OR "Support vector machine*" OR "Recurrent neural network*" OR "convolutional neural network*" OR "Artificial neural network*" OR "Deep neural network*" OR "Naïve Bayes" OR "Naive Bayes" OR "Random Forest" OR "Long Short-Term Memory Networks" OR "Gradient Boost*" OR AdaBoost OR XGboost OR "Multilayer Perceptron" OR "Ensemble learning" OR "Autoencoder" OR "Predict* model*") | 100 |
