# Supplementary material for: Performance of AI in Predicting the Progression of Gestational Diabetes to Type 2 Diabetes: Systematic Review and Meta-Analysis
Source: J Med Internet Res. 2026 Jul 9;28:e87882. doi: 10.2196/87882 (PMC13349230; doi:10.2196/87882)
Supplement: Multimedia Appendix 3 [file jmir-v28-e87882-s003.docx]

**Multimedia Appendix 3: Data Extraction Form**

| **Extracted data** | **Definition** |
| --- | --- |
| **Study Characteristics** |  |
| Author | The surname of the first author of the study. |
| Year of publication | The year in which the study was published. |
| Type of publication | The venue where the study was published: peer-reviewed journal articles, conference proceedings, or dissertations. |
| Country of publication | The country where the study was conducted (where was the data collected). |
| Study design | Did the study use retrospective or prospective design? |
| Follow-up duration | The length of time participants were monitored to determine whether they developed T2DM/PreDM. |
| **Participants Characteristics** |  |
| Data sources | What is the source of data that was used for developing the algorithms: Closed source (collected by authors/ not available online) , Open source ( public databases ) |
| Dataset name (if public dataset) | Name of dataset |
| Number of participants | Total number of participants used to train and test the model. |
| Number of cases | Number of participants diagnosed with T2DM/PreDM |
| Number of controls | Number of participants diagnosed without T2DM/PreDM |
| Mean age | What is the mean age of the participants in the study? |
| Mean BMI | What is mean BMI of the participants in the study? |
| Reference standard | How the actual status (T2DM/PreMed) of the participants was identified (How the study identified participants with/without T2DM/PreMed) |
| Guidelines used | The clinical guidelines used to diagnose women (e.g., ADA, WHO) |
| **AI** **Characteristics** |  |
| AI algorithm used | What are the AI algorithms/models (e.g., RF, SVM, ANN, CNN, RNN, DNN, k-NN, MLP, DBN, DBM, DPN BN, CRT, DT, LASSO, LR, MFA, MLR, MDL, NB, NN, NSC, RBFN) used in the paper? |
| Best-performing AI model/ final model used | AI model that achieved the highest performance in the study and is considered the primary model for reporting results. |
| Target condition | The AI model used to predict T2DM or/and PreMD. |
| Number of features | How many features/variables/ predictors were used to develop the model? |
| Features used | What are the features/variables/ predictors used for developing the models? |
| Input feature categories | Categorize these features into the following gorups: Anthropometric data, Clinical data, Demographic data, Laboratory data, Omics data |
| Significant features | Key variables/features that the AI model identified as most important for making predictions or classifications |
| Type of validation | What is the approach that was used to validate the developed algorithm (e.g., Training-test split, K-fold cross-validation, Nested Cross-Validation, Leave One Out cross-validation, Apparent validation, external validation)? |
| Performance metrics | What are metrics used to assess the preformance of AI model in the paper: Area Under the Curve (AUC), Sensitivity (Recall), Specificity, F1-score, Accuracy, etc.. |
