# Supplementary material for: Performance of AI in Predicting the Progression of Gestational Diabetes to Type 2 Diabetes: Systematic Review and Meta-Analysis
Source: J Med Internet Res. 2026 Jul 9;28:e87882. doi: 10.2196/87882 (PMC13349230; doi:10.2196/87882)
Supplement: Multimedia Appendix 4 [file jmir-v28-e87882-s004.docx]

**Multimedia Appendix 4: Risk of Bias Assessment Tool (PROBAST+AI)**

Answers to signalling questions in general:

- **Yes (Y):**
Clear, explicit evidence in the paper/report shows the criterion is fully met (with sufficient detail).

- **Probably Yes (PY):**
The paper does not state everything explicitly, but based on context or indirect evidence, it’s reasonable to assume the criterion was met.

- **No (N):**
Clear evidence that the criterion was *not* met, or it was done inappropriately (e.g., outcome not assessed consistently, predictors unavailable at time of intended use).

- **Probably No (PN):**
Reporting is incomplete, but given what’s described, it’s more likely the criterion was not met.

- **No Information (NI):**
The paper gives no usable information on this point, and you cannot reasonably infer it.

- All signaling questions are phrased so that “yes” indicates low ROB and “no” high ROB.
- The ratings PY and PN are included to allow judgments to be made when information is not sufficient to be confident in answering Y or N.
- Responses of Y are intended to have similar implications to responses of PY (and likewise for N and PN) but allow a distinction between something that is known and something that is likely to be the case.
- Assessors should use NI only when there is truly no information to answer a signaling question.

| **1. Participants** | **Signaling questions** | **Explanation** |
| --- | --- | --- |
|  | 1.1 Were appropriate data sources used? |  **Y**: The data origin is clearly traceable. There are sufficient details about how data were collected, what measurement procedures were used, and how participants were sampled.   **PY**: The data origin is more likely traceable. It is clear from the context and indirect evidence how data were collected, what measurement procedures were used, and how participants were sampled.   **N/PN**: The data origin is not traceable. There are insufficient details about how data were collected, what measurement procedures were used, and how participants were sampled.   **NI**: There is truly no information on the data sources to answer a signaling question. |
|  | 1.2 Was an appropriate study design used? |  **Y**: It is explicitly stated that it used prospective longitudinal cohort design.   **PY**: It is more likely that it used prospective longitudinal cohort design.   **N**: It is clearly stated that it used retrospective design or existing sourses (existing cohort studies or routine care registries).   **PN**: It is more likely that it used retrospective design or existing sourses (existing cohort studies or routine care registries).   **NI**: There is truly no information on the study design to answer a signaling question. |
|  | 1.3 Did the inclusions and exclusions of study participants result in a representative dataset? |  **Y**:   - The study explicitly describes inclusion and exclusion criteria. - There is no evidence of selective inclusion or exclusion, especially of subgroups (e.g., by BMI, **singleton pregnancies**, ethnicity, age, comorbidities). For example, excluding participants that were difficult to diagnose or from marginalised subgroups, or including participants already known to have the outcome at the time of the predictor measurements.    **PY**: The inclusion/exclusion criteria are not fully described, but:   - It’s reasonable to infer that the participants were representative. - There's no red flag suggesting inappropriate exclusions.    **N**:   - The study explicitly excluded important subgroups without justification. For example, excluding women with obesity, Polycystic Ovary Syndrome (PCOS), older age, non-White ethnicities, or only including women with a specific lab value or complication. - Participants were selected in a biased way (e.g., convenience sampling of low-risk women only).    **PN**:   - The reporting is incomplete or vague. - Based on what is described, we suspect exclusions or biases, but they are not made explicit. - The study uses data from a selective database, or lacks key subgroups, and does not justify why.    **NI**: The study provides no usable information on pariticpants inclusion and exclusion criteria, and no reasonable inference can be drawn. |
|  | **Risk-of-bias judgement:** Could the selection of participants have introduced bias? | - **Low risk of bias**: If the answer to all signaling questions is ‘Yes’ or ‘PY’  - **High risk of bias**: If the answer to any of the signaling questions is “No or PN”.  - **Unclear risk of bias**: If the answer to all signaling questions is “NI”.  Or if the answer to some signaling questions is “NI” and none of the answers to signaling questions is “No/PN”. |
|  | **Concerns regarding applicability:** Are there concerns that the included participants and data sources do not match the review question or the assessor’s intended use of the prediction model? |  **Low concern**: Participant population clearly matches the intended review question (i.e., Women with GDM regardless of its age, ethnicity, parity, comorbidity, etc.).   **High concern**: Participants that limit the study applicability to the review question or assessor’s intended use of the model. For example, studies that recruite pregnant women with and without GDM.   **Unclear concern**: Ther is no enough information about the participants and the data sources to judge thier applicability to the review question. |
| **2. Predictors** | 2.1 Were predictors defined and assessed in a similar way for all participants? |  **Y**: The study clearly states that all predictors were measured for all participants:   - Using the same methods (e.g., all participants had BMI calculated using weight/height measured at the same visit). - At the same time point (e.g., 6 weeks postpartum). - By the same personnel or trained staff, or at least following the same protocol - Using same thresholds for categorization.    **PY**: The reporting is not fully detailed, but:   - It is reasonable to assume that predictors were measured consistently (e.g., data is from an EHR or national registry with uniform data collection). - There is no indication of variability across participants.    **N**: It is explicitly that predictors were defined or measured differently across participants or subgroups. For example,   - Some participants had lab-based fasting glucose; others self-reported. - Some predictors were measured by different people with no standardization or validation. - Predictors were not measured at the same time point, or using different instruments. - Certain subgroups were assessed differently (e.g., race-based reference values without justification).    **PN**: Reporting is **incomplete**, and there are **indications** that predictors might not have been consistently defined or measured. For example:   - The data source is **heterogeneous** (multiple clinics or datasets). - Predictor timing is vague (e.g., "data from postpartum visits" without clarifying whether timing varied). - Predictor assessment seems **prone to variation**, but the paper does not mention standardization.    **NI**: There is no usable information on how predictors were defined or measured, and when or by whom predictors were assessed. Further, we cannot infer consistency from the study setting. |
|  | 2.2 Was any pre-processing of predictors similar for all participants? |  **Y**: The study clearly describes pre-processing procedures, and the same procedures were applied uniformly to all participants and all subgroups. If multiple sites were involved, they standardized pre-processing steps.   **PY**: The study does not fully describe pre-processing, but we can reasonably infer consistency (e.g., centralized dataset, single hospital, or well-known public preprocessing pipelines).   **N**: The study explicitly applied different pre-processing steps across sites, subgroups, data modalities (e.g., some participants had normalized lab values, others did not).  There is clear inconsistency in how predictors were processed (e.g., handling of missing data, scaling, encoding, or feature extraction).   **PN**: Pre-processing is poorly described, and the study involved multiple datasets, centers, or sources, and it is likely that pre-processing differed.  Or the predictors include complex data (e.g., images or text) with no details on standardization.   **NI**: The paper gives no usable information about pre-processing, and we cannot infer anything about consistency from context. |
|  | 2.3 Were predictor assessments made without knowledge of outcome data? |  **Y**:   - The study clearly states that predictor assessors were blinded to outcome data. - Or the predictors were explicitly collected before the outcome occurred in the prospective cohort studies. - Or it is retrospective study    **PY**: The study does not explicitly mention blinding, but:   - Predictors were clearly collected before outcome development. - Or predictors are objective and automated (e.g., lab tests, EHR data at baseline). - Or the study setting makes it very unlikely that outcome data was known during predictor assessment.    **N**:   - The study explicitly states that predictor assessors knew the outcome at the time of assessment. - Or predictors were collected or re-evaluated after the outcome occurred, and no blinding was applied. Especially problematic for subjective predictors (e.g., re-reading imaging after knowing who developed T2D).    **PN**:   - Reporting is vague, and we suspect that outcome status was known during predictor assessment. - Or predictor data was extracted retrospectively without mention of blinding. Especially if subjective judgment was used (e.g., manual coding, re-reading notes/ images).    **NI**: The study gives no information at all about timing of predictor vs. outcome assessment and whether blinding was used. And we cannot infer it from study design. |
|  | 2.4 Were the predictors available at the time the model was intended to be used? |  **Y**:   - The study clearly describes that all predictors used in the final model are available at the intended time of use. - Or the model is intended to be used at a certain point (e.g., postpartum), and all predictors are collected by that time.    **PY**: The intended use time is clear, and it seems reasonable to assume that all predictors were available by that time, even if not all are explicitly stated. For example, if the model is to be used at the time of delivery, and predictors include standard antenatal clinical measures (e.g., BMI, glucose, blood pressure), but the exact timing is not specified.   **N**: The model explicitly includes predictors that are only available **after** the intended use time. For example, using **postpartum data** in a model intended to be used **at GDM diagnosis**.   **PN**: The timing of model use is stated (e.g., postpartum or during pregnancy), but it's not clear whether some predictors were available at that time. There are time-sensitive variables, but the study doesn't explain when those predictors were measured. Especially when longitudinal or follow-up variables are used without clear timestamps.   **NI**: The paper provides no information on when the model is intended to be used or when the predictors were measured. And we can’t reasonably infer the timing from the context. |
|  | **Risk-of-bias assessment:** | - **Low risk of bias**: If the answer to all signaling questions is ‘Yes’ or ‘PY’  - **High risk of bias**: If the answer to any of the signaling questions is “No or PN”.  - **Unclear risk of bias**: If the answer to all signaling questions is “NI”.  Or if the answer to some signaling questions is “NI” and none of the answers to signaling questions is “No/PN”. |
|  | **Concerns regarding applicability:** Concern that definition, pre-processing, or timing of predictors does not match the review question/intended use. |  **Low concern**: Predictor definitions, assessment, pre-processing, and timing match the intended use of the model.   **High concern**: Predictor definition, assessment, pre-processing, or timing differs from the intended use.   **Unclear concern**: The study does not report sufficient details on predictor definition, assessment, pre-processing, or timing. And we cannot infer applicability from the study context. |
| **3. Outcome** | 3.1 Were outcomes defined and assessed appropriately? |  **Y**:   - 2-hour 75 grams Oral Glucose Tolerance Test (2h 75g-OGTT): Anytime after 4 weeks of delivery. Prediabetes: 140-199 mg/dL, Diabetes: ≥ 200 mg/dL - Or Fasting Plasma Glucose (FPG): Anytime after 6 weeks of delivery. Prediabetes: 100–125 mg/dL (5.6–6.9 mmol/L). Diabetes: ≥126 mg/dL (7.0 mmol/L), - Or Hemoglobin A1c (HbA1c): Anytime after 24 weeks (6 months) of delivery. Prediabetes: 5.7% to 6.4%. Diabetes: ≥ 6.5%. - Or the study clearly mentioned that they followed guideliens recommended by American Diabetes Association (ADA), American College of Obstetricians and Gynecologists (ACOG), or the World Health Organization (WHO).    **PY**: Although not all details are explicitly reported, the context suggests outcomes were likely defined and assessed according to appropriate standards. For example, the study cites guideline-based definitions or established protocols but omits some operational details.   **N**: there is explicit evidence shows that outcome definitions or assessment methods were inappropriate. Examples include use of arbitrary investigator-chosen thresholds, reliance on less accurate or subjective measures (e.g., self-reported diabetes without verification), use of test that is inapprorpaite for the postpartum follow-up. For example, using HbA1C to assess DM after 10 weeks of delivery.   **PN**: Reporting is incomplete, but what is described suggests outcomes were more likely assessed inappropriately. For instance, outcomes were drawn from routine care or administrative databases without sufficient clarification.   **NI**: The study provides no usable information on outcome definitions or assessment methods, and no reasonable inference can be drawn. |
|  | 3.2 Was the outcome defined and determined in a similar way for all participants? |  **Y**: There is clear, explicit evidence that outcomes were defined and measured uniformly across all participants, regardless of setting, subgroup, or predictor test results. For example, all women are assessed for T2DM using the same prespecified standard (e.g., fasting glucose or HbA1c at defined timepoints) with consistent thresholds.   **PY**: Although not all details are explicitly stated, it is reasonable to assume that outcomes were assessed consistently. For example, the study describes guideline-based criteria but omits minor procedural details (e.g., timing of follow-up visits) without suggesting differences across participants.   **N**: Evidence shows that outcomes were not assessed in a similar way across all participants. Examples include:   - Different definitions or thresholds applied across sites or subgroups. - Partial verification, where only participants who self-reported that they have DM undergo reference standard assessment. - Differential verification, where some groups are evaluated with less accurate methods (e.g., confirmatory glucose test for one group but self-report for another).    **PN**: Reporting is incomplete, but given the description, it is more likely that outcome assessment varied across participants. For example, outcomes are drawn from heterogeneous registries or databases without harmonization, or assessment methods are implied to differ by subgroup.   **NI**: The study provides no usable information on whether outcomes were assessed consistently, and no reasonable inference can be drawn. |
|  | 3.3 Was the outcome determined without knowledge of predictor data? |  **Y**:   - The study clearly states that outcome assessors were blinded to predictor data. - Or the outcome was determined using objective laboratory measures (e.g., fasting glucose, HbA1c) without reference to the predictors included in the model. - There is clear, explicit evidence that outcome assessments were performed independently of predictor information.    **PY**: The study does not fully describe blinding or masking procedures, but based on context it is reasonable to assume that predictor data were not used in outcome assessments. For instance, outcomes are based on standardized diagnostic criteria from lab tests, even if blinding is not explicitly reported.   **N**: Evidence shows that outcome assessment was influenced by predictor data. For example, predictor variables (e.g., BMI, blood pressure) explicitly included in the outcome definition.   **PN**: Reporting is incomplete, but what is described suggests that predictor data were likely incorporated into outcome assessment.   **NI**: The study provides no usable information on whether outcome assessors had access to predictor data, and no reasonable inference can be drawn. |
|  | **3.4** Was the time interval between predictor assessment and outcome assessment appropriate? |  **Y**: There is explicit evidence that the outcome was assessed after 3-120 months of delivery.   **PY**: The study does not fully specify the timing but provides enough context to reasonably assume the interval is appropriate (3-120 months). For instance, outcomes are reported at “regular postpartum visits” without exact duration, but the follow-up period appears aligned with disease progression.   **N**: There is explicit evidence that the time interval was clearly inappropriate (<3 months or > 120 months).   **PN**: Reporting is incomplete, but what is described suggests the interval is likely inappropriate. For example, studies relying on administrative data without specifying the time gap, where outcomes may have been missed or measured long after predictor assessment.   **NI**: The study provides no usable information on the time interval between predictor and outcome assessment, and no reasonable inference can be drawn. |
|  | **Risk-of-bias assessment** | - **Low risk of bias**: If the answer to all signaling questions is ‘Yes’ or ‘PY’  - **High risk of bias**: If the answer to any of the signaling questions is “No or PN”.  - **Unclear risk of bias**: If the answer to all signaling questions is “NI”.  Or if the answer to some signaling questions is “NI” and none of the answers to signaling questions is “No/PN”. |
|  | **Concerns regarding applicability:** Are there concerns that the outcome definition, timing, or determination do not match the review question? |  **Low concern**: Outcome definition, measurement, and timing align with review question.   **High concern**: Outcome definition, measurement, or timing does not match review question.   **Unclear concern**: The study does not report sufficient details on outcome definition, assessment, or timing. And we cannot infer applicability from the study context. |
| **4.** **Analysis:** | 4.1 Was model evaluation based on only apparent performance avoided? |  **Y**: There is clear, explicit evidence that apparent performance alone was avoided. The study reports internal validation (e.g., Hold-out, K-fold CV, Leave One Out Cross validation) and/or external validation using a separate dataset.   **PY**: The study does not explicitly state that apparent performance was avoided, but based on context it is reasonable to assume internal or external validation was performed (e.g., mentions “test set” or “cross-validation” without full detail).   **N**: Evidence shows that model performance was evaluated only on the same dataset used for development, without any form of validation. Examples: reporting only accuracy, AUC, or other performance metrics from the development dataset without resampling, splitting, or external validation.   **PN**: Reporting is incomplete, but what is described suggests that only apparent performance was assessed. For example, results are presented without clarity on whether resampling or independent data were used, and performance metrics align suspiciously with training data.   **NI**: The study provides no usable information on how model performance was evaluated, and no reasonable inference can be drawn. |
|  | 4.2 Was there evidence that the sample size was reasonable? |  **Y**: There is clear, explicit evidence that the sample size was adequate to produce precise estimates. Or authors followed established sample size guidance for validation. Or number of events per predictor parameter (EPP) ≥ 10.   **PY**: The study does not provide full details, but context suggests the sample was likely sufficient. For example, several hundred participants with a reasonable number of events are reported, even if explicit justification is missing.   **N**: Evidence shows that the the sample was clearly too small to yield reliable performance estimates. Or number of events per predictor parameter (EPP) < 10.   **PN**: Reporting is incomplete, but available information suggests the evaluation sample was likely too small. Examples include:  • Very low numbers of outcome events (e.g., <20 cases of T2DM).  • Extremely unbalanced case–control ratios.   **NI**: The study provides no usable information on sample size, number of events, or effective sample size in subgroups, and no reasonable inference can be drawn. |
|  | 4.3 Were participants with missing or censored data handled appropriately? |  **Y**: There is clear evidence that there is no missing data. Or Clear evidence that missing and/or censored data were handled appropriately. Examples include:   - Use of multiple imputation approaches or other accepted statistical approaches. - Explicit justification that the chosen approach reflects intended real-world use (e.g., evaluating model robustness in routine EHR data with expected missingness).    **PY**: The study does not mention whether there is missing data. Or handling of missing or censored data is not fully described, but context suggests it was likely appropriate.   **N**: Evidence shows missing or censored data were handled inappropriately. Examples include:   - Excluding all participants with any missing predictor or outcome values (complete-case analysis) without justification. - Omitting predictors from a model during external validation because they were missing in that dataset.    **PN**: Reporting is incomplete, but what is described suggests inappropriate handling of missing data. For example, missing data are acknowledged, and there are unjustified exclusions, but the study did not mention how the missing data were handled.   **NI**: The study provides no usable information on how missing or censored data were handled, and no reasonable inference can be drawn. |
|  | 4.4 If methods to address class imbalance were used, was the evaluation done in a dataset without imbalance correction?* |  **Y**: There is clear, explicit evidence that any imbalance correction methods (if used) were applied only during model development. Performance evaluation was carried out on unaltered, representative data with the true event prevalence.   **PY**: Reporting does not explicitly confirm this, but context suggests evaluation was done on data without imbalance correction. For example, the study states that oversampling was used for training but refers to a separate “test set” or “validation cohort” without imbalance correction.   **N**: Evidence shows imbalance correction was also applied to the evaluation/test dataset. For example, resampling techniques were applied directly to the whole dataset or test/validation dataset.   **PN**: Reporting is incomplete, but what is described suggests that imbalance correction may have been applied to the evaluation data. For instance, the study reports performance on a “balanced dataset” without clarifying whether this balancing occurred only during training.   **NI**: The study provides no usable information on whether imbalance correction was avoided in the evaluation dataset, and no reasonable inference can be drawn. |
|  | 4.5 If data splitting was done to create training and test datasets, was there evidence that data leakage avoided?* |  **Y**: There is clear, explicit evidence that data leakage was avoided. For example:   - Training, validation, and test datasets are strictly separated. - Preprocessing (e.g., normalization, feature selection, imputation) is applied only within the training set and then transferred to the test set. - Hyperparameter tuning was done exclusively in the training/validation phase, not on the test set.    **PY**: Leakage is not explicitly ruled out, but context suggests it was avoided. For instance, the study mentions an independent test set and standard cross-validation without detailed explanation of preprocessing steps.   **N**: Clear evidence of data leakage. Examples include:   - Preprocessing or feature selection applied to the entire dataset before splitting. - Hyperparameters tuned directly on the test set. - Duplicate or overlapping records between training and evaluation sets.    **PN**: Reporting is incomplete, but available information suggests leakage likely occurred. For instance, vague methods like “data were normalized prior to splitting” without clarification, or ambiguous descriptions of how validation was handled.   **NI**: The study provides no usable information on how data splitting and preprocessing were conducted, and no reasonable inference can be drawn. |
|  | **4.6** If resampling methods were used to evaluate model performance, were all model development steps replicated in the resampling process?* |  **Y**: There is clear, explicit evidence that all model development steps were included in each resampling iteration. For example:   - Authors state that preprocessing, imputation, feature selection, and model training were repeated within each fold of cross-validation. - Hyperparameter tuning was conducted strictly within training folds, not on the full dataset.    **PY**: The report does not fully describe all steps, but context suggests appropriate replication. For instance, the study describes nested cross-validation for hyperparameter tuning but does not explicitly mention imputation handling.   **N:** Evidence shows that not all steps were replicated. Examples include:   - Imputation or feature selection applied to the full dataset before resampling. - Hyperparameter tuning performed on the complete dataset, then resampling only applied to final model fitting. - Only the model training step was repeated, while preprocessing was done once.    **PN**: Reporting is incomplete, but the description suggests improper replication. For instance, the paper states “cross-validation was applied after feature selection” without clarification that selection was repeated within folds.   **NI**: The study provides no usable information about whether all development steps were included in the resampling procedure, and no reasonable inference can be drawn. |
|  | **Risk-of-bias assessment:** Could the analysis, its conduct, or its interpretation have introduced bias? | - **Low risk of bias**: If the answer to all signaling questions is ‘Yes’ or ‘PY’  - **High risk of bias**: If the answer to any of the signaling questions is “No or PN”.  - **Unclear risk of bias**: If the answer to all signaling questions is “NI”.  Or if the answer to some signaling questions is “NI” and none of the answers to signaling questions is “No/PN”. |

OVERALL JUDGEMENT

- **Risk of Bias**
- Low → if all 4 domains are low.
- High → if any domain is high.
- Unclear → if ≥1 unclear & none high.
- **Applicability**
- Low → if all domains are low concern.
- High → if ≥1 high concern.
- Unclear → if ≥1 unclear & none high.

**Mapping of CHARMS Checklist to PROBAST+AI Domains**

| **CHARMS Domain** | **Mapped PROBAST+AI domain** | **How addressed in this review** |
| --- | --- | --- |
| **Source of Data** | Participants | Assessed under participant selection and data source appropriateness |
| **Participants** | Participants | Evaluated through signalling questions on inclusion/exclusion and representativeness |
| **Outcome to be Predicted** | Outcome | Assessed via outcome definition, measurement consistency, and blinding |
| **Candidate Predictors** | Predictors | Evaluated through predictor definition, preprocessing, and availability at prediction time |
| **Sample Size** | Analysis | Assessed through adequacy of sample size and risk of overfitting |
| **Missing Data** | Analysis | Evaluated based on reporting and handling of missing data |
| **Model Development** | Analysis | Assessed through modelling methods, feature selection, and analytical rigor |
| **Model Performance** | Analysis | Evaluated via reported performance metrics (e.g., AUC, sensitivity, specificity) |
| **Model Evaluation** | Analysis | Assessed via validation strategy (e.g., cross-validation, lack of external validation) |
| **Results** | Analysis | Evaluated through completeness of reported model outputs |
| **Interpretation & Discussion** | Not directly assessed | - |
