# Supplementary material for: Performance of AI in Predicting the Progression of Gestational Diabetes to Type 2 Diabetes: Systematic Review and Meta-Analysis
Source: J Med Internet Res. 2026 Jul 9;28:e87882. doi: 10.2196/87882 (PMC13349230; doi:10.2196/87882)
Supplement: Multimedia Appendix 5 [file jmir-v28-e87882-s005.docx]

**Multimedia Appendix 5: Characteristics of the included studies**

| Study [Ref] | Year | Publication type | Country | Study Type | Follow-up Duration (months) |
| --- | --- | --- | --- | --- | --- |
| Allalou[1] | 2016 | Journal article | United States | Retrospective | 48 |
| Chung [2] | 2025 | Journal article | Sweden | Retrospective | 120 |
| Ilari [3] | 2022 | Journal article | Austria | Retrospective | 84 |
| Joglekar[4] | 2021 | Journal article | Australia | Prospective | 120 |
| Khan [5] | 2019 | Journal article | United States | Retrospective | 24 |
| Krishnan [6] | 2019 | Conference paper | India | Prospective | 120 |
| Lai [7] | 2020 | Journal article | United States | Retrospective | 96 |
| Lin [8] | 2011 | Journal article | Taiwan | Prospective | NR |
| Parkhi [9] | 2023 | Journal article | United Kingdom | Retrospective | 3.25 |
| Prashanthan [10] | 2025 | Journal article | United Kingdom | Prospective | NR |
| NR: not reported | | | | | |

1. Allalou, A., et al., *A Predictive Metabolic Signature for the Transition From Gestational Diabetes Mellitus to Type 2 Diabetes.* Diabetes, 2016. **65**(9): p. 2529-39.

2. Chung, H.S., et al., *Longitudinal clinical and proteomic diabetes signatures in women with a history of gestational diabetes.* JCI Insight, 2025. **10**(3).

3. Ilari, L., et al., *Unraveling the Factors Determining Development of Type 2 Diabetes in Women With a History of Gestational Diabetes Mellitus Through Machine-Learning Techniques.* Front Physiol, 2022. **13**: p. 789219.

4. Joglekar, M.V., et al., *Postpartum circulating microRNA enhances prediction of future type 2 diabetes in women with previous gestational diabetes.* Diabetologia, 2021. **64**(7): p. 1516-1526.

5. Khan, S.R., et al., *The discovery of novel predictive biomarkers and early-stage pathophysiology for the transition from gestational diabetes to type 2 diabetes.* Diabetologia, 2019. **62**(4): p. 687-703.

6. Krishnan, D.R., et al., *Evaluation of predisposing factors of Diabetes Mellitus post Gestational Diabetes Mellitus using Machine Learning Techniques*, in *2019 IEEE Student Conference on Research and Development (SCOReD)*. 2019, IEEE: Seri Iskandar, Perak, Malaysia.

7. Lai, M., et al., *Amino acid and lipid metabolism in post-gestational diabetes and progression to type 2 diabetes: A metabolic profiling study.* PLoS Med, 2020. **17**(5): p. e1003112.

8. Lin, H.C., C.T. Su, and P.C. Wang, *An application of artificial immune recognition system for prediction of diabetes following gestational diabetes.* J Med Syst, 2011. **35**(3): p. 283-9.

9. Parkhi, D., et al., *Prediction of postpartum prediabetes by machine learning methods in women with gestational diabetes mellitus.* iScience, 2023. **26**(10): p. 107846.

10. Prashanthan, J. and A. Prashanthan, *Predicting the future risk of developing type 2 diabetes in women with a history of gestational diabetes mellitus using machine learning and explainable artificial intelligence.* Prim Care Diabetes, 2025.
