# Supplementary material for: Performance of AI in Predicting the Progression of Gestational Diabetes to Type 2 Diabetes: Systematic Review and Meta-Analysis
Source: J Med Internet Res. 2026 Jul 9;28:e87882. doi: 10.2196/87882 (PMC13349230; doi:10.2196/87882)
Supplement: Multimedia Appendix 6 [file jmir-v28-e87882-s006.docx]

**Multimedia Appendix 6: Characteristics of participants**

| Study [Ref] | Data Source | Number of Participants | Number of Cases | Number of Controls | Mean Age | BMI | Reference Standard | Guidelines |
| --- | --- | --- | --- | --- | --- | --- | --- | --- |
| Allalou[1] | Closed | 244 | 122 | 122 | 33.9 | 33.2 | 2h 75g OGTT, EHRs | ADA |
| Chung [2] | Closed | 206 | 79 | 127 | 33.4 | 26.1 | 2h 75g OGTT, FPG, HbA1C, Self-reported | WHO |
| Ilari [3] | Closed | 75 | 17 | 58 | 34 | 26.6 | IM-IVGTT | NR |
| Joglekar[4] | Closed | 103 | 21 | 82 | 31.9 | 25.9 | 2h 75g OGTT | ADA |
| Khan [5] | Closed | 140 | 55 | 85 | 34.5 | 32.3 | 2h 75g OGTT | NR |
| Krishnan [6] | Closed | 77 | 17 | 60 | NR | NR | Self-reported | NR |
| Lai [7] | Closed | 658 | 173 | 485 | 33 | 32.1 | 2h 75g OGTT, EHRs | ADA |
| Lin [8] | Closed | 152 | 42 | 110 | NR | NR | 2h 75g OGTT | National guidelines |
| Parkhi [9] | Closed | 394 | 92 | 302 | 32.2 | 29.7 | 2h 75g OGTT, HbA1C | ADA |
| Prashanthan [10] | Closed | 6000 | NR | NR | NR | NR | NR | NR |
| ADA: American Diabetes Association; BMI: Body Mass Index; EHRs: Electronic Health Records; FPG: Fasting Plasma Glucose; HbA1C: Glycated Hemoglobin; IM-IVGTT: Insulin Modified Intravenous Glucose Tolerance Test; NR: not reported; OGTT: Oral Glucose Tolerance Test; WHO: World Health Organization | | | | | | | | |

1. Allalou, A., et al., *A Predictive Metabolic Signature for the Transition From Gestational Diabetes Mellitus to Type 2 Diabetes.* Diabetes, 2016. **65**(9): p. 2529-39.

2. Chung, H.S., et al., *Longitudinal clinical and proteomic diabetes signatures in women with a history of gestational diabetes.* JCI Insight, 2025. **10**(3).

3. Ilari, L., et al., *Unraveling the Factors Determining Development of Type 2 Diabetes in Women With a History of Gestational Diabetes Mellitus Through Machine-Learning Techniques.* Front Physiol, 2022. **13**: p. 789219.

4. Joglekar, M.V., et al., *Postpartum circulating microRNA enhances prediction of future type 2 diabetes in women with previous gestational diabetes.* Diabetologia, 2021. **64**(7): p. 1516-1526.

5. Khan, S.R., et al., *The discovery of novel predictive biomarkers and early-stage pathophysiology for the transition from gestational diabetes to type 2 diabetes.* Diabetologia, 2019. **62**(4): p. 687-703.

6. Krishnan, D.R., et al., *Evaluation of predisposing factors of Diabetes Mellitus post Gestational Diabetes Mellitus using Machine Learning Techniques*, in *2019 IEEE Student Conference on Research and Development (SCOReD)*. 2019, IEEE: Seri Iskandar, Perak, Malaysia.

7. Lai, M., et al., *Amino acid and lipid metabolism in post-gestational diabetes and progression to type 2 diabetes: A metabolic profiling study.* PLoS Med, 2020. **17**(5): p. e1003112.

8. Lin, H.C., C.T. Su, and P.C. Wang, *An application of artificial immune recognition system for prediction of diabetes following gestational diabetes.* J Med Syst, 2011. **35**(3): p. 283-9.

9. Parkhi, D., et al., *Prediction of postpartum prediabetes by machine learning methods in women with gestational diabetes mellitus.* iScience, 2023. **26**(10): p. 107846.

10. Prashanthan, J. and A. Prashanthan, *Predicting the future risk of developing type 2 diabetes in women with a history of gestational diabetes mellitus using machine learning and explainable artificial intelligence.* Prim Care Diabetes, 2025.
