# Supplementary material for: Performance of AI in Predicting the Progression of Gestational Diabetes to Type 2 Diabetes: Systematic Review and Meta-Analysis
Source: J Med Internet Res. 2026 Jul 9;28:e87882. doi: 10.2196/87882 (PMC13349230; doi:10.2196/87882)
Supplement: Multimedia Appendix 7 [file jmir-v28-e87882-s007.docx]

**Multimedia Appendix 7: Characteristics of AI Algorithms**

**Part 1**

| Study [Ref] | AI Model | Best Performing AI Model | Target Condition | Number of Features | Type of Validation | ML performances Metrices |
| --- | --- | --- | --- | --- | --- | --- |
| Allalou [1] | DT, LogReg, NB | DT | T2DM | 24 | Hold-out | Acc, AUC, F1, Sen, Spe |
| Chung [2] | GB, LogReg, RF | RF | T2D, PreDM | 477 | K-Fold CV | AUC |
| Ilari [3] | DT, LogReg, NB | LogReg | T2DM | 34 | K-Fold CV | Acc, AUC, F1, Pre, Sen, Spe |
| Joglekar [4] | LogReg | LogReg | T2DM | 10 | Hold-out | AUC, Sen, Spe |
| Khan [5] | DT | DT | T2DM | 626 | K-Fold CV | Acc, AUC, F1, Pre, Sen, Spe |
| Krishnan [6] | AdaBoost, DT, NB, RF, SVM | RF, NB | T2DM | 15 | K-Fold CV, | Acc, F1, Sen, Spe |
| Lai [7] | RF | RF | T2DM | 39 | Hold-out | Acc, AUC, F1, Pre, Sen, Spe |
| Lin [8] | AIRS, DT, LogReg, SVM | AIRS | T2D, PreDM | 12 | K-Fold CV | Sen |
| Parkhi [9] | Bagging classifier, CatBoost, DT, GB, LightGBM, LogReg, RF, XGBoost | LogReg | PreDM | 21 | Nested CV | Acc, AUC, F1, NPV, Pre |
| Prashanthan [10] | AdaBoost, Bagging classifier, CatBoost, DT, Extra Trees, GB, KNN, LDA, LightGBM, LogReg, MLP, NB, QDA, RF, Ridge, SVM, XGBoost | AdaBoost | T2DM | 28 | Hold-out | Acc, F1, Pre, Sen |
| Acc: Accuracy; AdaBoost: Adaptive Boosting; AI: Artificial Intelligence; AIRS: Artificial Immune Recognition System; AUC: Area Under the Curve; Bagging: Bootstrap Aggregating; CatBoost: Categorical Boosting; CV: Cross-Validation; DT: Decision Tree; F1: F1 Score; GB: Gradient Boosting; GDM: Gestational Diabetes Mellitus; KNN: K-Nearest Neighbors; LDA: Linear Discriminant Analysis; LightGBM: Light Gradient Boosting Machine; LogReg: Logistic Regression; MLP: Multi-Layer Perceptron; NB: Naïve Bayes; NPV: Negative Predictive Value; Pre: Precision; PreDM: Prediabetes; QDA: Quadratic Discriminant Analysis; RF: Random Forest; Sen: Sensitivity; Spe: Specificity; SVM: Support Vector Machine; T2D: Type 2 Diabetes; T2DM: Type 2 Diabetes Mellitus; XGBoost: Extreme Gradient Boosting | | | | | | |

**Part 2**

| Study [Ref] | Input Feature Categories | Features Used | Significant Features |
| --- | --- | --- | --- |
| Allalou [1] | Laboratory data, Omics data | Fasting plasma glucose (FPG), 2-hour Plasma Glucose (2-PG), 2-AAA, Gly, Ile, Leu, Thr, Trp, Tyr, Val, xLeu+, Hexoses, SM (OH) C16:1, SM (OH) C22:2, SM C18:0, SM C18:1, SM C20:2, SM C24:1, PC ae C40:5, PC ae C42:5, PC ae C44:5, AC10, AC3, Palmitoleic acid (C16:1 n9) | PC ae C40:5 (phosphatidylcholine), Hexoses, BCAAs (Valine, Leucine, Isoleucine), SM (OH) C14:1 (sphingomyelin), Fasting plasma glucose (FPG), 2-hour OGTT glucose (2hPG) |
| Chung [2] | Anthropometric data, Clinical data, Demographic data, Laboratory data, Omics data | Age, Weight, BMI, BP, Body fat, Waist-hip ratio, Glucose, Insulin, Cholesterol, T4, TSH, HDL, LDL, hs-CRP, Leptin, Adiponectin, HOMA-IR, HOMA-B, Proteomics | T2DM (PLTP, PON3, ITIH4, C3, SOD3, ALDOB, FUCA1, ITIH1, MASP2, HSPG2), PreDM (Fat mass, Waist, FCN2, BMI, Hip, TGFBI, Leptin, NOTCH3, GNPTG, Weight) |
| Ilari [3] | Anthropometric data, Demographic data, Laboratory data | Age, Weight, Height, BMI, Fasting plasma glucose (FPG), Mean area under the glucose curve, Mean area under the insulin curve, Mean area under the C-peptide curve, Area under the insulin curve during the 1st phase of test, Area under the insulin curve during the 2nd phase of test, Disappearance rate of glucose before insulin injection, Disappearance rate of glucose after insulin injection, Insulin sensitivity, Glucose effectiveness, Distribution volume of glucose, Basal insulin effect of glucose effectiveness, Glucose effectiveness at zero insulin, Mean of suprabasal insulin in the time interval 3–8 min, Mean of suprabasal C-peptide in the time interval 3–8 min, Disposition index, Basal secretion rate, β-cell responsivity to glucose, Area under the secretion curve during the entire test, Area under the secretion curve during the 1st phase of test, Area under the secretion curve during the 2nd phase of test, Mean insulin clearance during the entire test, Mean insulin clearance during the 1st phase of test, Mean insulin clearance during the 2nd phase of test, Extra-hepatic insulin clearance, Hepatic insulin clearance, Peak insulin after glucose injection, Peak insulin after insulin injection, Peak C-peptide, Glucose dose injected, | Age, BMI, Fasting plasma glucose (FPG), Disposition index, Basal secretion rate of insulin, Mean area under the C-peptide concentration curve |
| Joglekar [4] | Anthropometric data, Demographic data, Laboratory data, Omics data | Age, BMI, Pregnancy fasting glucose (FPG), Postnatal fasting glucose (FPG), Cholesterol, Triacylglycerol, miR-491-5p, miR-543, miR-410-3p, miR-369-3p | Age, BMI, Fasting plasma glucose (FPG), Postnatal fasting glucose, Cholesterol, Triacylglycerol, miR-369-3p |
| Khan [5] | Clinical data, Laboratory data, Omics data | Metabolites, Fasting plasma glucose (FPG), HOMA-IR, 2-hour Plasma Glucose (2-PG), TAGs, Family history of DM, Type of GDM treatment | seven specific lipid metabolites. |
| Krishnan [6] | Laboratory data | White Blood Cells, Neutrophils, Lymphocytes, Monocytes, Eosinophil, Basophil, Red Blood Cells Count, Hemoglobin, Hematocrit, Mean Corpuscular Value, Mean Corpuscular Hemoglobin, Mean Corpuscular Hemoglobin Concentration, Red Blood Cells Distribution Width, Platelet Count, Mean Platelet Volume | NR |
| Lai [7] | Laboratory data, Omics data | Fasting plasma glucose (FPG), 2-hour Plasma Glucose (2-PG), Hexose, Gln, Glu, His, Ile, Leu, Lys, Met, Pro, Ser, Thr, Trp, Tyr, AC10, AC16, AC3, Kynurenine, Spermidine, lysoPC a C17:0, lysoPC a C26:0, lysoPC a C26:1, PC aa C30:0, PC aa C32:1, PC aa C32:2, PC aa C36:2, PC aa C38:3, PC aa C40:4, PC ae C38:1, PC ae C38:4, PC ae C40:4, PC ae C40:5, PC ae C42:3, PC ae C42:5, PC ae C44:6, SM (OH) C16:1, SM (OH) C22:2, SM (OH) C24:1 | Hexose, His, Spermidine, AC10, Kynurenine, lysoPC a C26:0, total DMA, PC ae C40:4, Ser, Glu, PC aa C32:2, Tyr, PC aa C30:0, Ile, AC3, lysoPC a C26:1, SM (OH) C22:2, Gln, PC aa C32:1, SM C20:2 |
| Lin [8] | Anthropometric data, Clinical data, Demographic data, Laboratory data | Age, BMI, Weight, Increased weight during pregnancy, Weight of newborn, Family history of DM, Number of pregnancy times, Fasting plasma glucose (FPG), 1-hour Plasma Glucose (1-PG), 2-hour Plasma Glucose (2-PG), 3-hour Plasma Glucose (3-PG), 50-g oral glucose challenge test value, | NR |
| Parkhi [9] | Anthropometric data, Clinical data, Demographic data, Laboratory data | Age, Height, Weight, BMI, BP, Ethnicity, Parity, Smoking status, Married status, Employment status, Gestational age at delivery, Mode of delivery, Birth weight, Birth Centile, Breastfeeding status, Fasting plasma glucose (FPG), Antenatal postprandial glucose (A-PG), Antenatal HbA1c (A-HbA1c), Gestational age at antenatal OGTT | Fasting plasma glucose (FPG), Antenatal HbA1c |
| Prashanthan [10] | Anthropometric data, Clinical data, Demographic data, Laboratory data, Omics | Age, pre-pregnancy BMI, Parity, Ethnicity, Family history of DM, Type of GDM treatment, Hypertensive disorders during pregnancy, Preterm delivery, Postpartum hemorrhage, Gestational weight gain, OGTT results (2-PG & FPG), HbA1c level, Baby weight, Gestational age, Mode of delivery, Still birth/Miscarriage, History of Recurrence of GDM, NICU admission, Perinatal outcome, Deprivation quintile, Presence of T2DM-associated gene variants (e.g., TCF7L2, FTO), Postpartum weight gain, Physical activity, Diet, Smoking status, Alcohol intake, Postpartum glucose screening, Breastfeeding status | Type of GDM treatment, Physical inactivity, BMI, breastfeeding status, history of recurrent GDM, Parity, Diet, Ethnicity |
| 2hPG: 2-hour Oral Glucose Tolerance Test Glucose; 2-PG: 2-hour Plasma Glucose; 3-PG: 3-hour Plasma Glucose; AC3: Acylcarnitine C3; AC10: Acylcarnitine C10; A-HbA1c: Antenatal Hemoglobin A1c; A-PG: Antenatal Postprandial Glucose; ALDOB: Aldolase B; BCAA: Branched-Chain Amino Acids; BMI: Body Mass Index; BP: Blood Pressure; C3: Complement Component 3; DM: Diabetes Mellitus; FPG: Fasting Plasma Glucose; FCN2: Ficolin-2; FTO: Fat Mass and Obesity-Associated Gene; GDM: Gestational Diabetes Mellitus; GNPTG: N-Acetylglucosamine-1-Phosphate Transferase Gamma; Gln: Glutamine; Glu: Glutamate; HbA1c: Hemoglobin A1c; HDL: High-Density Lipoprotein; HOMA-B: Homeostatic Model Assessment for Beta-cell Function; HOMA-IR: Homeostatic Model Assessment for Insulin Resistance; hs-CRP: High-Sensitivity C-Reactive Protein; HSPG2: Heparan Sulfate Proteoglycan 2; Ile: Isoleucine; ITIH1: Inter-Alpha-Trypsin Inhibitor Heavy Chain H1; ITIH4: Inter-Alpha-Trypsin Inhibitor Heavy Chain H4; Kynurenine: Kynurenine (Tryptophan Metabolite); LDL: Low-Density Lipoprotein; Leu: Leucine; lysoPC: Lysophosphatidylcholine; MASP2: Mannan-Binding Lectin Serine Protease 2; Met: Methionine; miR: MicroRNA; NICU: Neonatal Intensive Care Unit; NOTCH3: Neurogenic Locus Notch Homolog Protein 3; OGTT: Oral Glucose Tolerance Test; PC aa: Phosphatidylcholine Diacyl; PC ae: Phosphatidylcholine Acyl-Alkyl; PON3: Paraoxonase 3; PreDM: Prediabetes; Pro: Proline; Ser: Serine; SM: Sphingomyelin; SM (OH): Hydroxysphingomyelin; SOD3: Superoxide Dismutase 3; TAG: Triacylglycerol; TCF7L2: Transcription Factor 7-Like 2; TGFBI: Transforming Growth Factor Beta-Induced Protein; Thr: Threonine; Trp: Tryptophan; TSH: Thyroid Stimulating Hormone; T4: Thyroxine; Tyr: Tyrosine; Val: Valine | | | |

1. Allalou, A., et al., *A Predictive Metabolic Signature for the Transition From Gestational Diabetes Mellitus to Type 2 Diabetes.* Diabetes, 2016. **65**(9): p. 2529-39.

2. Chung, H.S., et al., *Longitudinal clinical and proteomic diabetes signatures in women with a history of gestational diabetes.* JCI Insight, 2025. **10**(3).

3. Ilari, L., et al., *Unraveling the Factors Determining Development of Type 2 Diabetes in Women With a History of Gestational Diabetes Mellitus Through Machine-Learning Techniques.* Front Physiol, 2022. **13**: p. 789219.

4. Joglekar, M.V., et al., *Postpartum circulating microRNA enhances prediction of future type 2 diabetes in women with previous gestational diabetes.* Diabetologia, 2021. **64**(7): p. 1516-1526.

5. Khan, S.R., et al., *The discovery of novel predictive biomarkers and early-stage pathophysiology for the transition from gestational diabetes to type 2 diabetes.* Diabetologia, 2019. **62**(4): p. 687-703.

6. Krishnan, D.R., et al., *Evaluation of predisposing factors of Diabetes Mellitus post Gestational Diabetes Mellitus using Machine Learning Techniques*, in *2019 IEEE Student Conference on Research and Development (SCOReD)*. 2019, IEEE: Seri Iskandar, Perak, Malaysia.

7. Lai, M., et al., *Amino acid and lipid metabolism in post-gestational diabetes and progression to type 2 diabetes: A metabolic profiling study.* PLoS Med, 2020. **17**(5): p. e1003112.

8. Lin, H.C., C.T. Su, and P.C. Wang, *An application of artificial immune recognition system for prediction of diabetes following gestational diabetes.* J Med Syst, 2011. **35**(3): p. 283-9.

9. Parkhi, D., et al., *Prediction of postpartum prediabetes by machine learning methods in women with gestational diabetes mellitus.* iScience, 2023. **26**(10): p. 107846.

10. Prashanthan, J. and A. Prashanthan, *Predicting the future risk of developing type 2 diabetes in women with a history of gestational diabetes mellitus using machine learning and explainable artificial intelligence.* Prim Care Diabetes, 2025.
