# Supplementary material for: Performance of AI in Predicting the Progression of Gestational Diabetes to Type 2 Diabetes: Systematic Review and Meta-Analysis
Source: J Med Internet Res. 2026 Jul 9;28:e87882. doi: 10.2196/87882 (PMC13349230; doi:10.2196/87882)
Supplement: Multimedia Appendix 8 [file jmir-v28-e87882-s008.docx]

**Multimedia Appendix 8: Reviewers’ judgments about each domain in risk of bias and applicability concerns for each included study**
