# Supplementary material for: Performance of AI in Predicting the Progression of Gestational Diabetes to Type 2 Diabetes: Systematic Review and Meta-Analysis
Source: J Med Internet Res. 2026 Jul 9;28:e87882. doi: 10.2196/87882 (PMC13349230; doi:10.2196/87882)

**Figure S1.** Forest plot of the highest accuracy for type 2 diabetes mellitus for the best performing artificial intelligence model in each study. ML: machine learning; T2DM: type 2 diabetes mellitus.


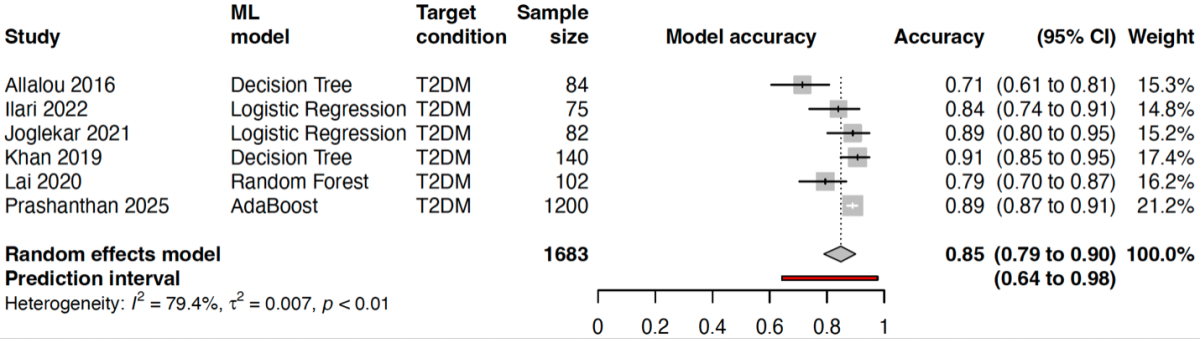


**Figure S2.** Forest plot of the highest accuracy for type 2 diabetes mellitus for the worst performing artificial intelligence model in each study. ML: machine learning; T2DM: type 2 diabetes mellitus.


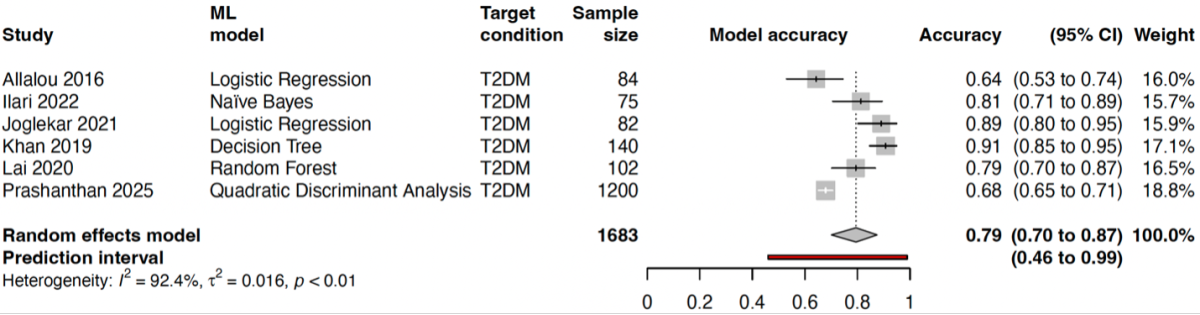


**Figure S3.** Forest plot of accuracy for type 2 diabetes mellitus by artificial intelligence model type. T2DM: type 2 diabetes mellitus.


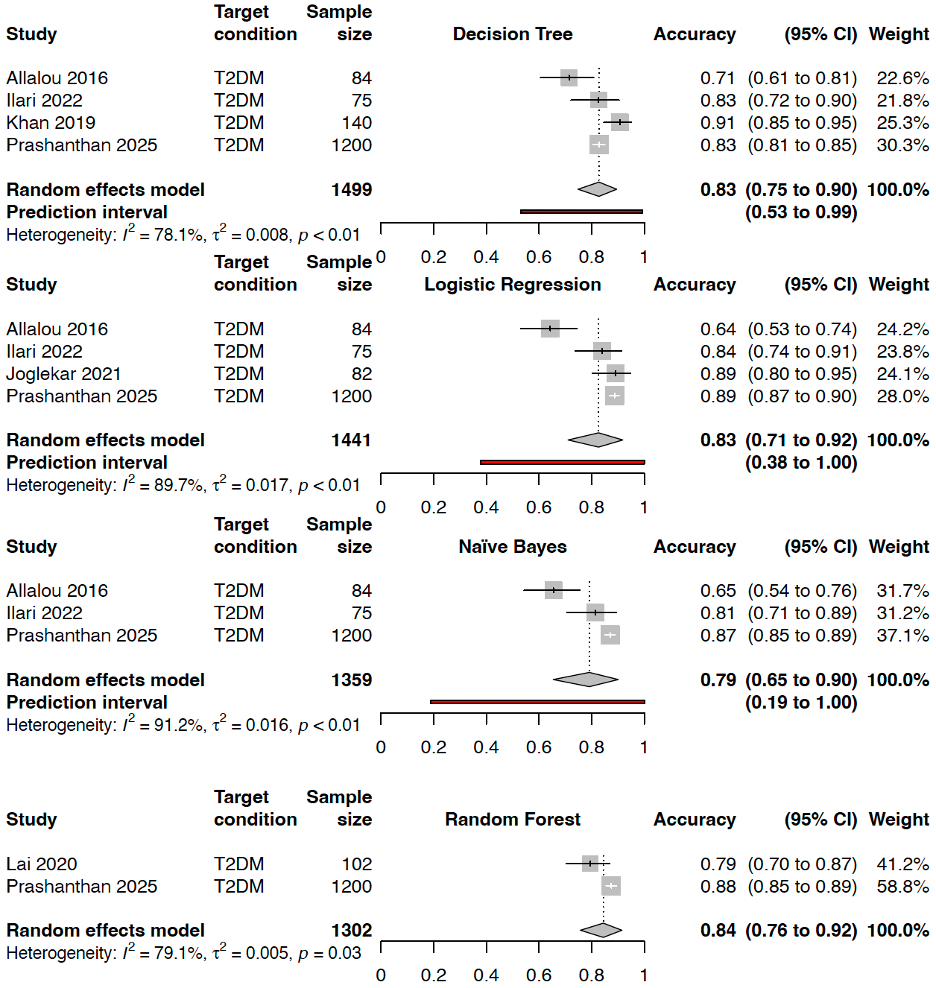


**Figure S4.** Forest plot of the highest sensitivity for type 2 diabetes mellitus for the best performing artificial intelligence model in each study. T2DM: type 2 diabetes mellitus.


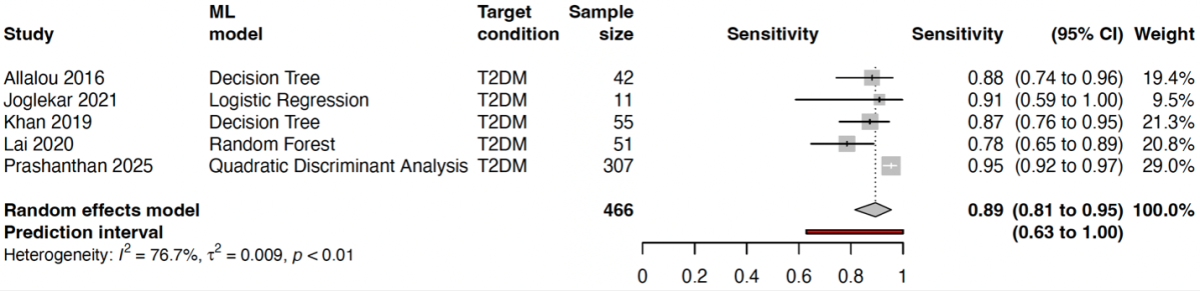


**Figure S5.** Forest plot of the highest sensitivity for type 2 diabetes mellitus for the worst performing artificial intelligence model in each study. ML: machine learning; T2DM: type 2 diabetes mellitus.


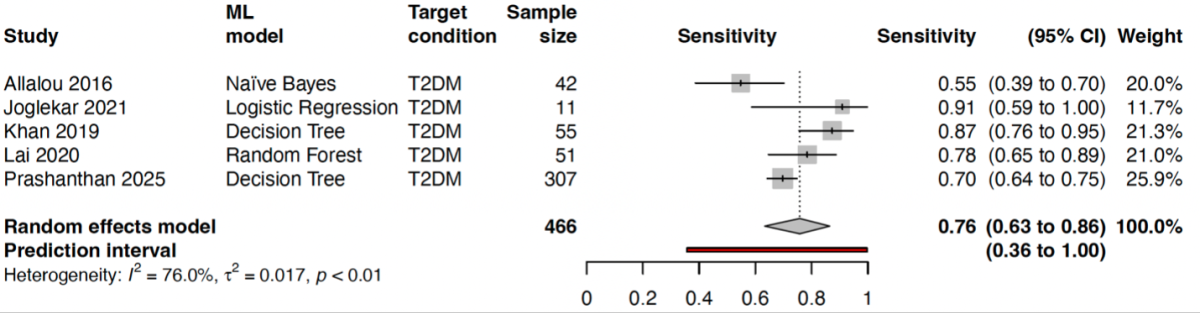


**Figure S6.** Forest plot of sensitivity for type 2 diabetes mellitus by artificial intelligence model. T2DM: type 2 diabetes mellitus.


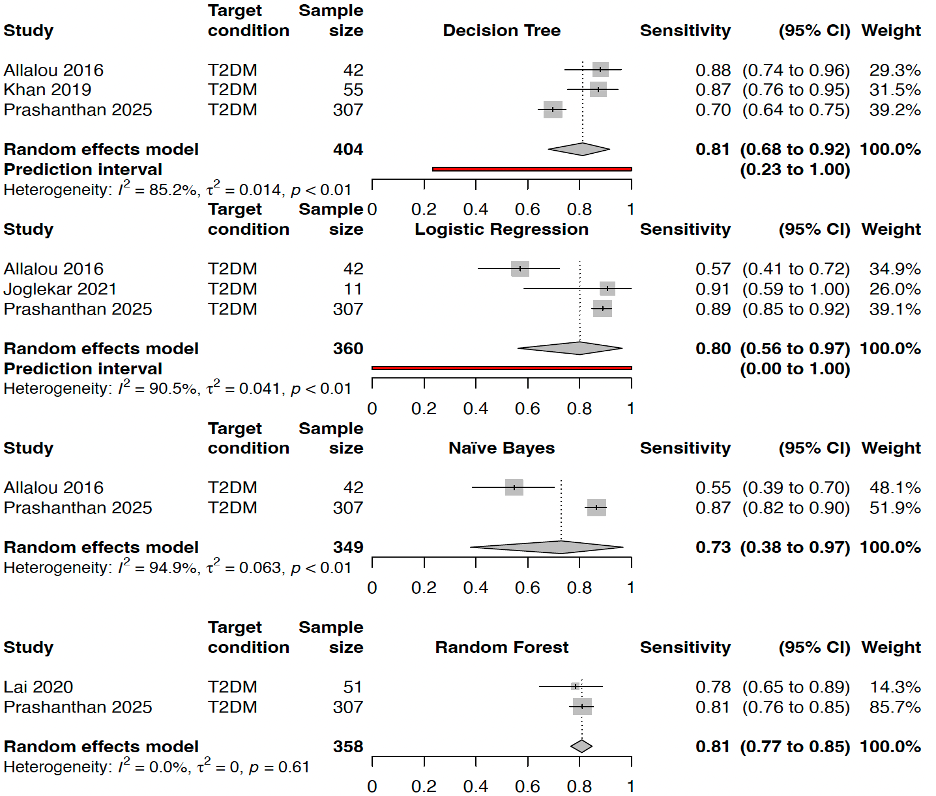


**Figure S7.** Forest plot of the highest specificity for type 2 diabetes mellitus for the best performing artificial intelligence model in each study. ML: machine learning; T2DM: type 2 diabetes mellitus.


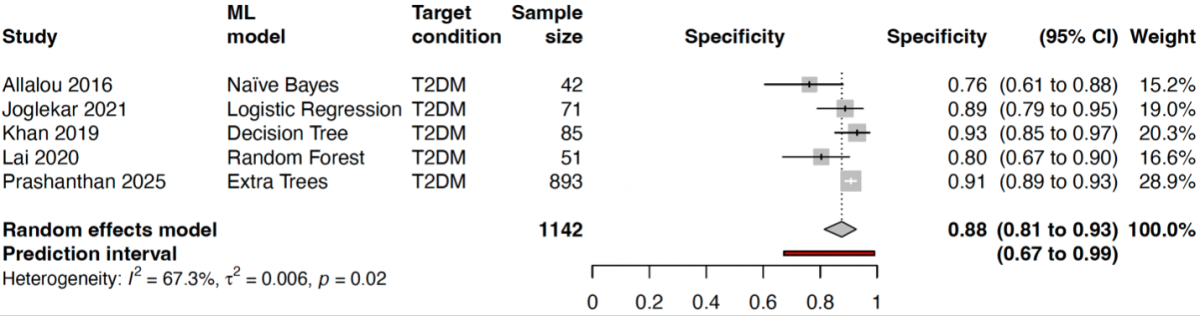


**Figure S8.** Forest plot of the highest specificity for type 2 diabetes mellitus for the worst performing artificial intelligence model in each study. ML: machine learning; T2DM: type 2 diabetes mellitus.


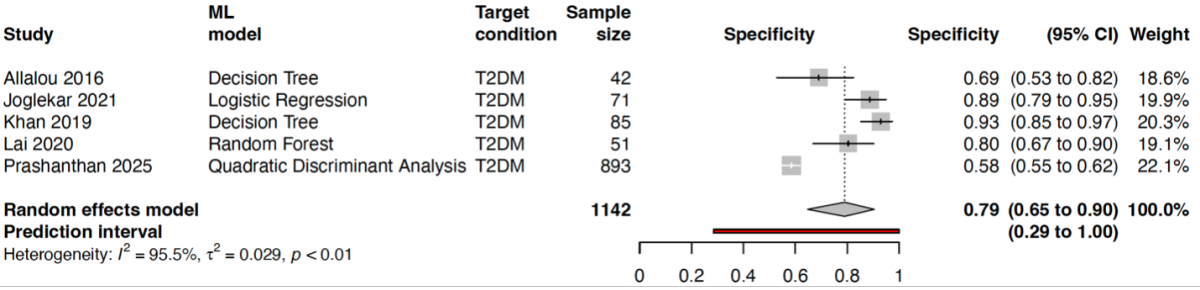


**Figure S9.** Forest plot of specificity for type 2 diabetes mellitus by artificial intelligence model. T2DM: type 2 diabetes mellitus.


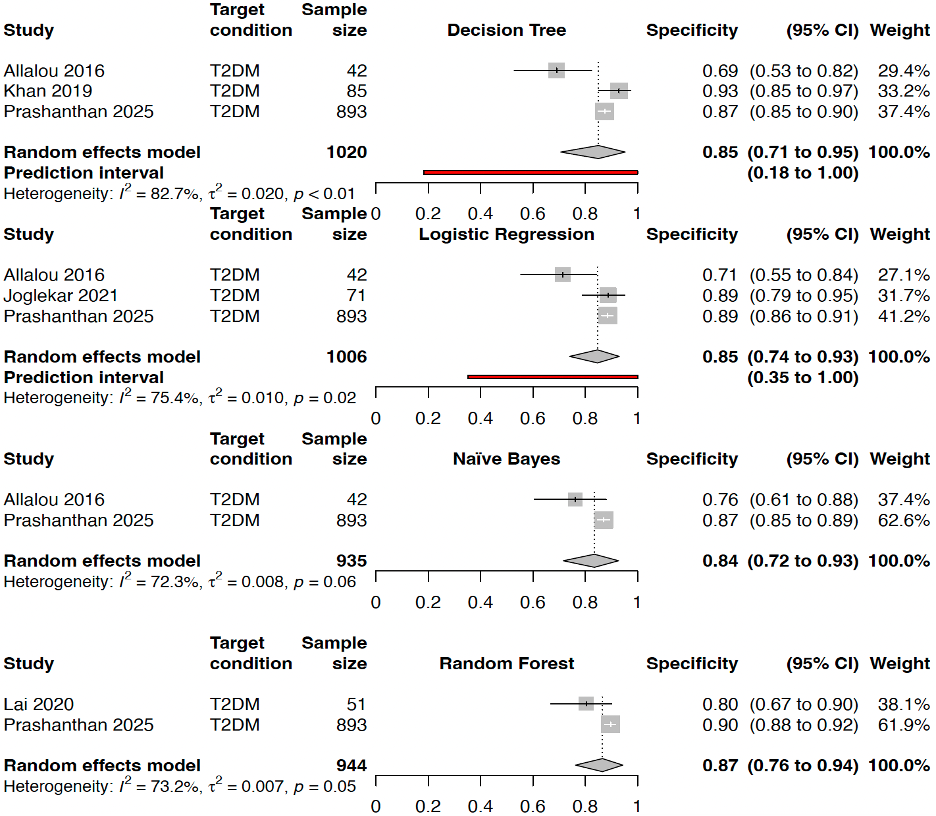


**Figure S10.** Forest plot of the highest *F*_1_-scores for type 2 diabetes mellitus for the best performing artificial intelligence model in each study. T2DM: type 2 diabetes mellitus.


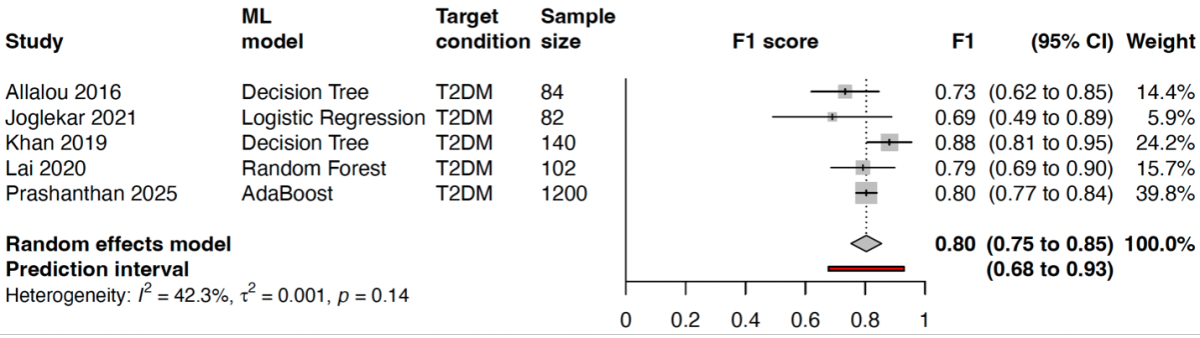


**Figure S11.** Forest plot of the highest *F*_1_-scores for type 2 diabetes mellitus for the worst performing artificial intelligence model in each study. ML: machine learning; T2DM: type 2 diabetes mellitus.


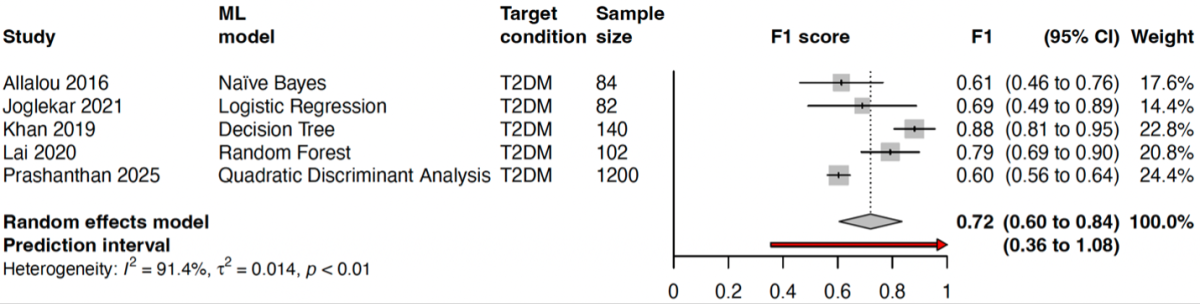


**Figure S12.** Forest plot of *F*_1_-score for type 2 diabetes mellitus by artificial intelligence model type. ML: machine learning; T2DM: type 2 diabetes mellitus.


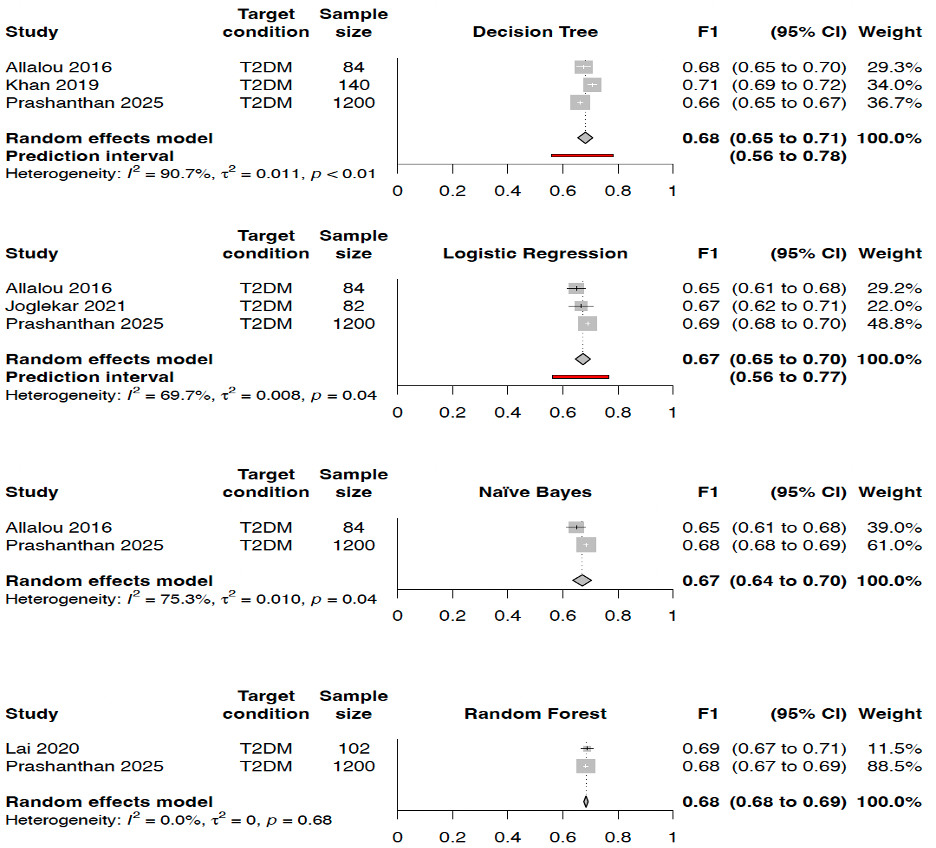


**Figure S13.** Forest plot of the highest area under the curve for type 2 diabetes mellitus for the best performing artificial intelligence model in each study. AUC: area under the curve; ML: machine learning; T2DM: type 2 diabetes mellitus.


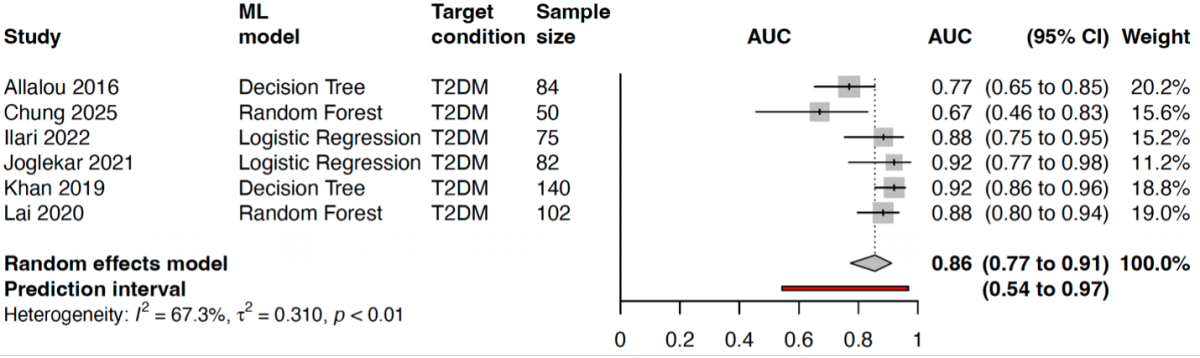


**Figure S14.** Forest plot of the highest area under the curve for type 2 diabetes mellitus for the worst performing artificial intelligence model in each study. AUC: area under the curve.; ML: machine learning; T2DM: type 2 diabetes mellitus.


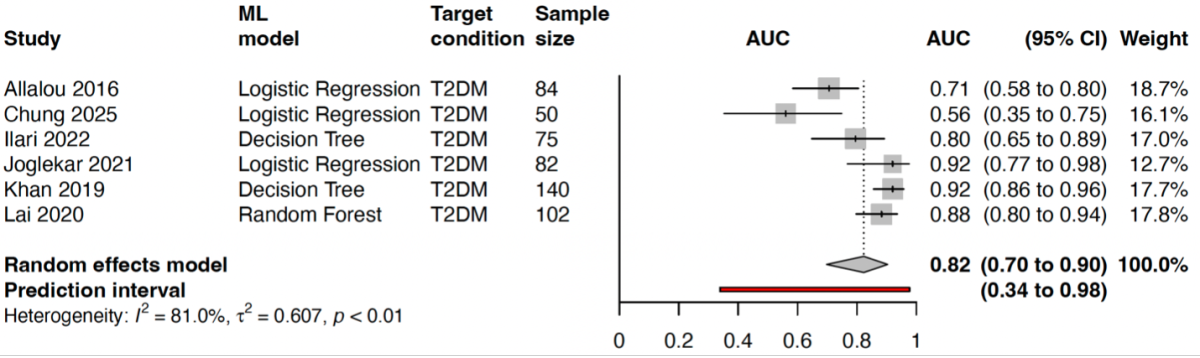


**Figure S15.** Forest plot of area under the curve for type 2 diabetes mellitus by artificial intelligence model type. AUC: area under the curve; T2DM: type 2 diabetes mellitus.


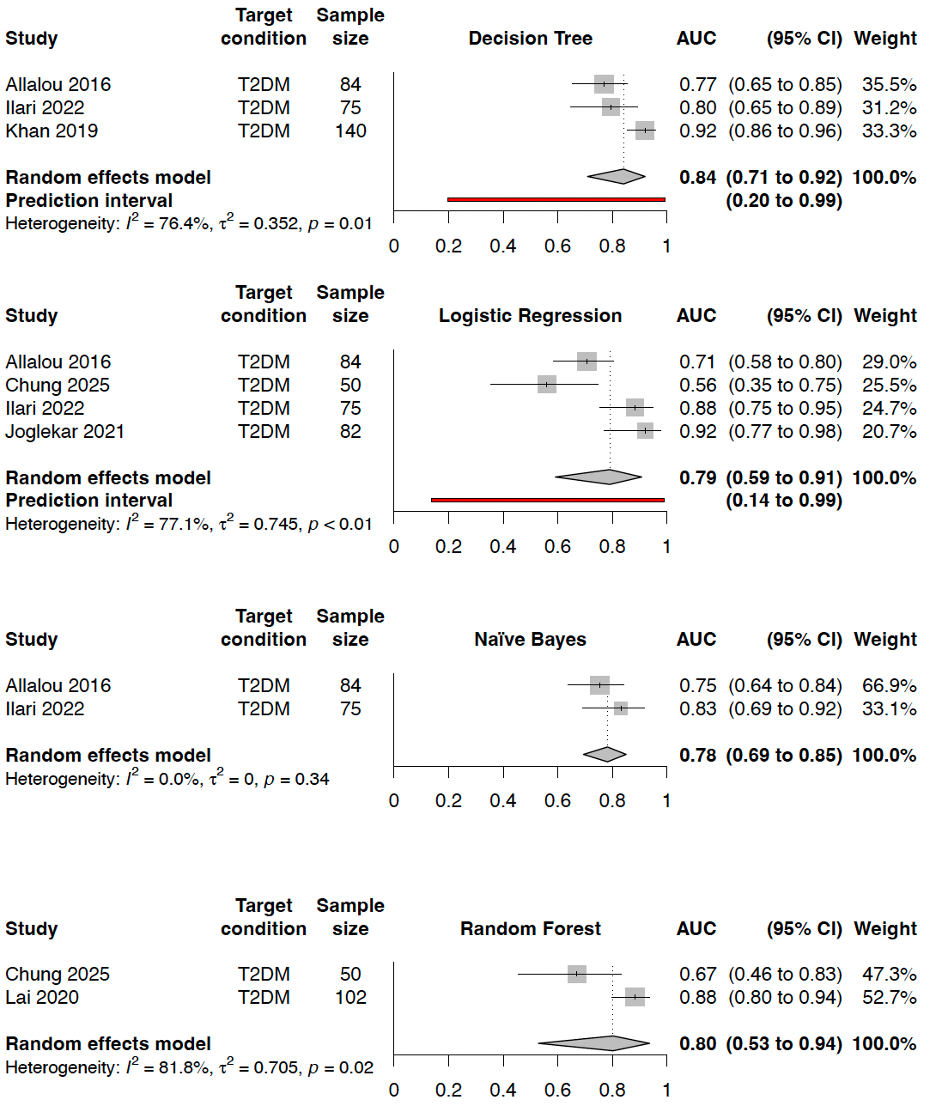


**Figure S16.** Forest plot of the highest area under the curve for prediabetes for the best performing artificial intelligence model in each study. AUC: area under the curve; ML: machine learning; PreDM: prediabetes.


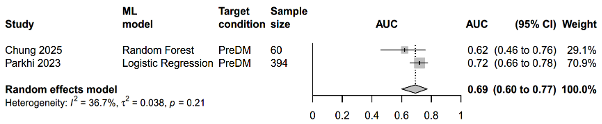


**Figure S17.** Forest plot of the highest area under the curve for prediabetes for the worst performing artificial intelligence model in each study. AUC: area under the curve; ML: machine learning; PreDM: prediabetes.


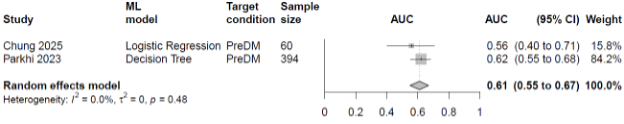


**Figure S18.** Forest plot of area under the curve for prediabetes by artificial intelligence model type. AUC: area under the curve; PreDM: prediabetes.


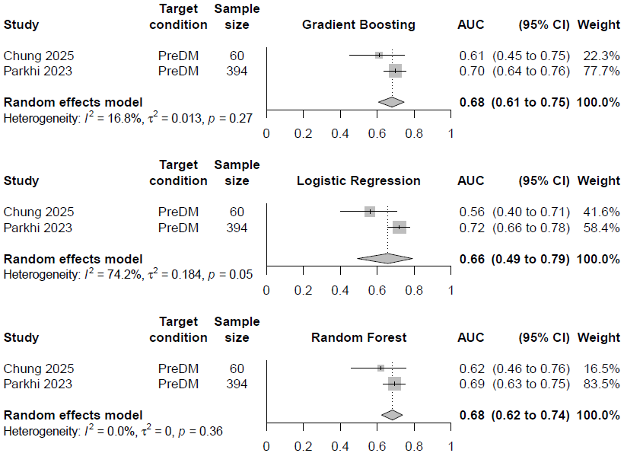

Supplement: Multimedia Appendix 10 [file jmir-v28-e87882-s010.docx]
