# Supplementary material for: Performance of AI in Predicting the Progression of Gestational Diabetes to Type 2 Diabetes: Systematic Review and Meta-Analysis
Source: J Med Internet Res. 2026 Jul 9;28:e87882. doi: 10.2196/87882 (PMC13349230; doi:10.2196/87882)
Supplement: Multimedia Appendix 13 [file jmir-v28-e87882-s013.docx]

**Multimedia Appendix 12 Forest plots for F1 score**

**
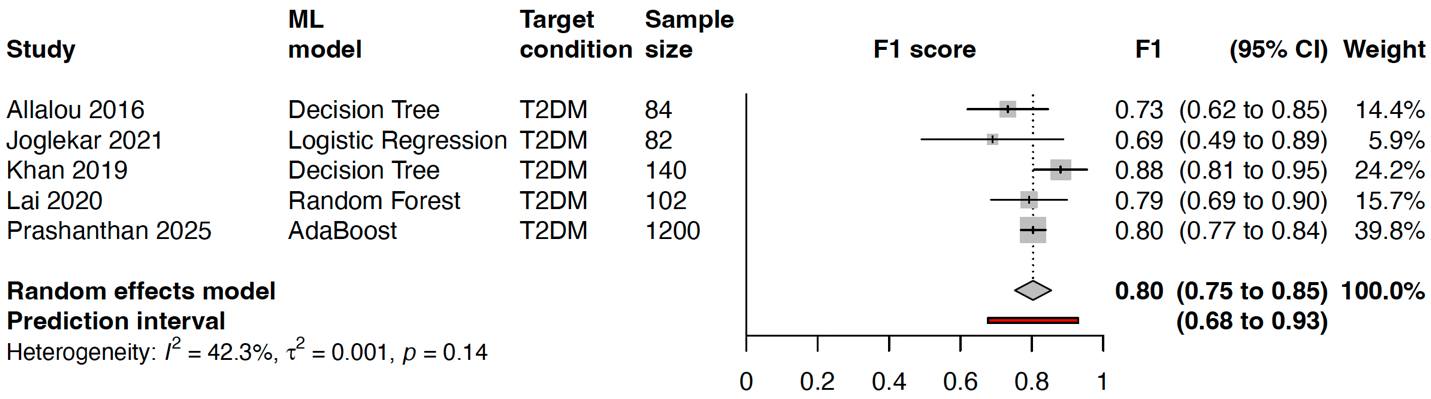
**

**Supplemental Figure 1** Forest plot of the highest F1 scores for T2DM for the best performing AI model in each study

**
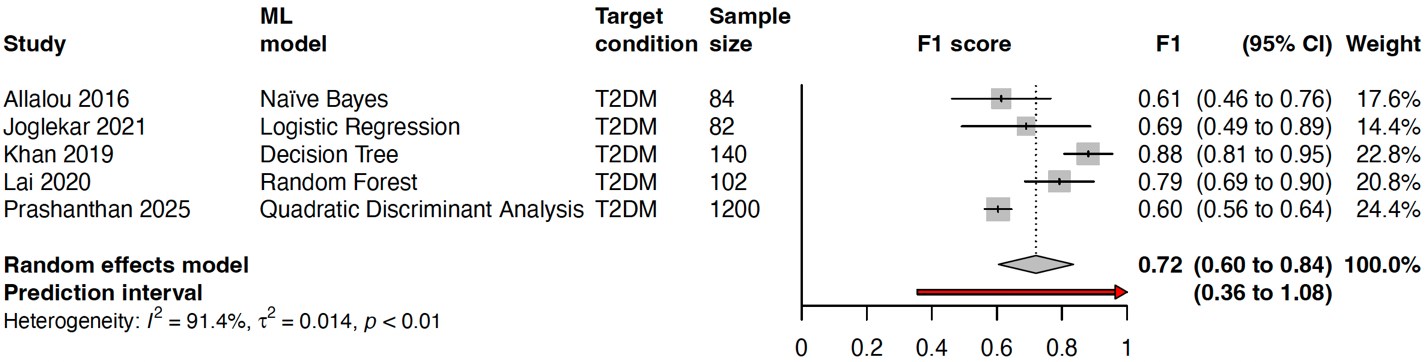
**

**Supplemental Figure 2** Forest plot of the highest F1 scores for T2DM for the worst performing AI model in each study

**
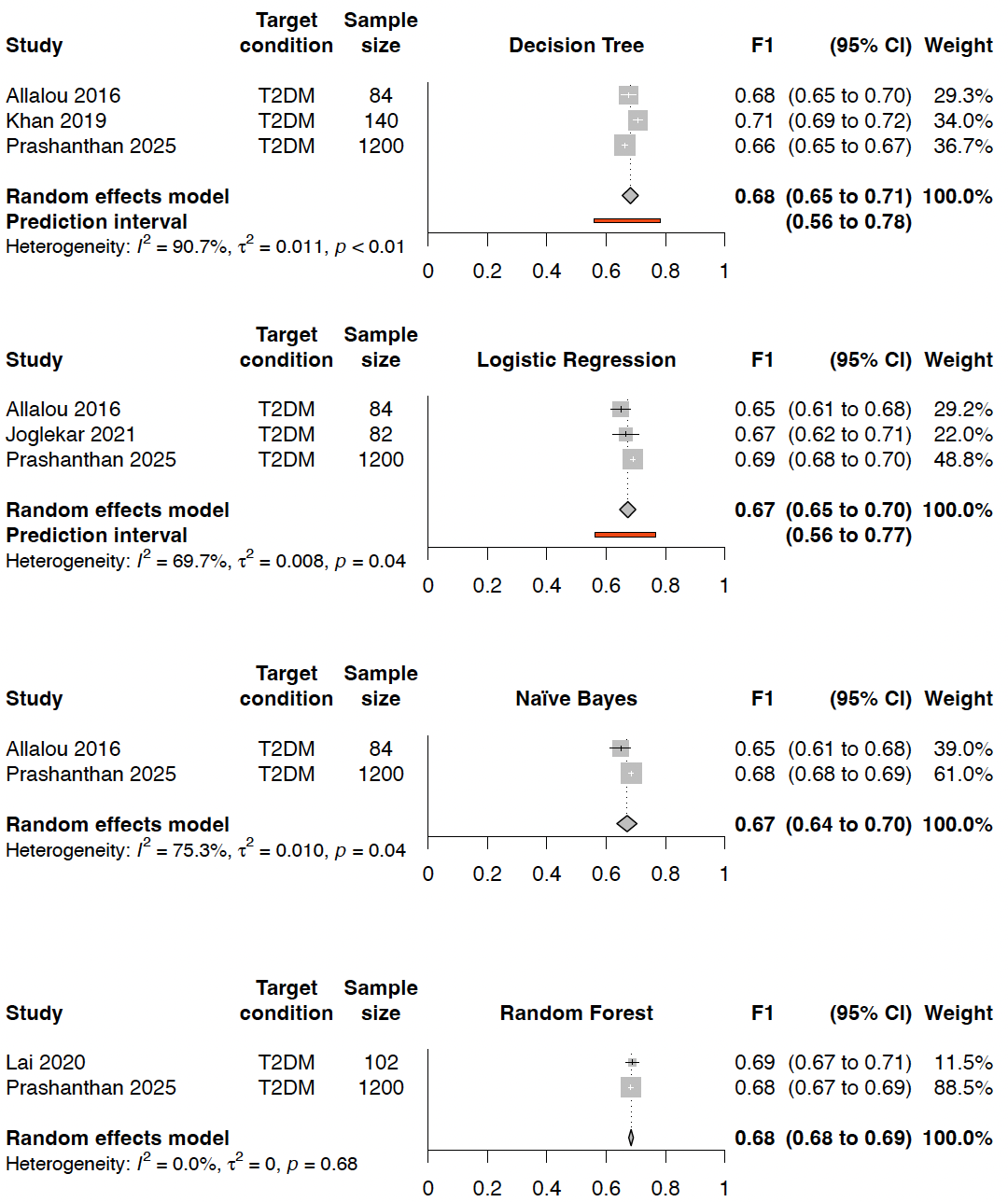
**

**Supplemental Figure 3** Forest plot for F1 scores for T2DM by AI model type
