# Supplementary material for: Performance of AI in Predicting the Progression of Gestational Diabetes to Type 2 Diabetes: Systematic Review and Meta-Analysis
Source: J Med Internet Res. 2026 Jul 9;28:e87882. doi: 10.2196/87882 (PMC13349230; doi:10.2196/87882)
Supplement: Multimedia Appendix 15 [file jmir-v28-e87882-s015.docx]

Prashanthan 2025

0.02

0.01

0.00

Khan 2019

0.04

0.03

Lai 2020

0.05

Allalou 2016


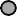

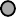

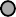

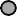

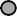

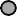


Standard Error

Joglekar 2021

Ilari 2022

Prashanthan 2025

0.00


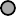

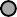

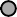

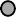

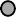

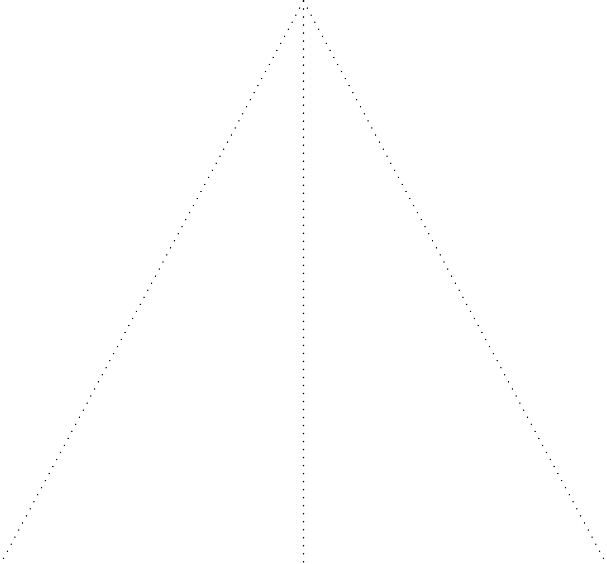


Standard Error

Lai 2020

0.10

0.05

Khan 2019

Allalou 2016

Joglekar 2021

0.15

Prashanthan 2025

0.00


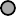

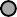

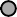

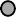

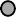

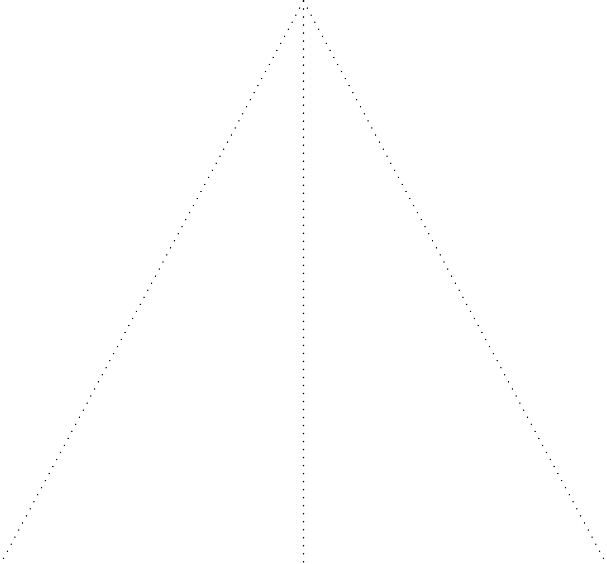


Standard Error

Lai 2020

0.10

0.05

Khan 2019

Allalou 2016

Joglekar 2021

0.15

Standard Error

Prashanthan 2025


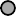

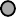

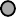

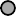

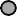

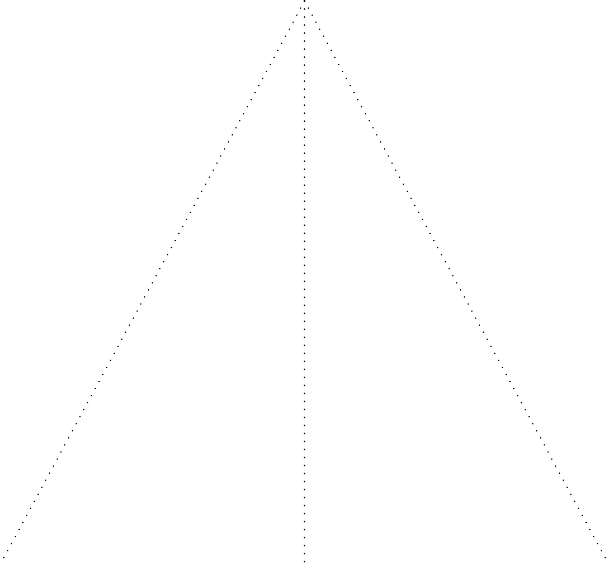


0.02

0.00

Khan 2019

0.04

Lai 2020

0.06

Allalou 2016

0.08

Joglekar 2021

0.10

0.6 0.7 0.8 0.9 1.0

Allalou 2016


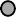

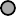

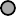

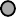

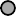

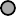

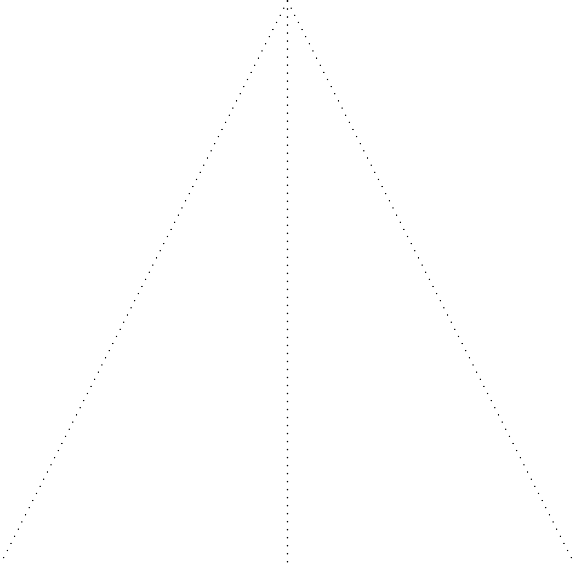


0.3

0.2

0.1

0.0

Lai 2020

Khan 2019

Chung 2025

0.5

0.4

Ilari 2022

Joglekar 2021

Standard Error

0.5 1.0 1.5 2.0 2.5 3.0

0.6

Logit transformed AUC
